# Extracellular signal regulated kinase 5 promotes cell migration, invasion and lung metastasis in a FAK-dependent manner

**DOI:** 10.1007/s13238-020-00701-1

**Published:** 2020-03-06

**Authors:** Weiwei Jiang, Fangfang Cai, Huangru Xu, Yanyan Lu, Jia Chen, Jia Liu, Nini Cao, Xiangyu Zhang, Xiao Chen, Qilai Huang, Hongqin Zhuang, Zi-Chun Hua

**Affiliations:** 1 The State Key Laboratory of Pharmaceutical Biotechnology, College of Life Sciences, Nanjing University, Nanjing, 210023 China; 2 Changzhou High-Tech Research Institute of Nanjing University and Jiangsu TargetPharma Laboratories Inc., Changzhou, 213164 China

**Keywords:** ERK5, lung cancer, melanoma, metastasis, FAK, USF1, EMT

## Abstract

This study was designed to evaluate ERK5 expression in lung cancer and malignant melanoma progression and to ascertain the involvement of ERK5 signaling in lung cancer and melanoma. We show that ERK5 expression is abundant in human lung cancer samples, and elevated ERK5 expression in lung cancer was linked to the acquisition of increased metastatic and invasive potential. Importantly, we observed a significant correlation between ERK5 activity and FAK expression and its phosphorylation at the Ser^910^ site. Mechanistically, ERK5 increased the expression of the transcription factor USF1, which could transcriptionally upregulate FAK expression, resulting in FAK signaling activation to promote cell migration. We also provided evidence that the phosphorylation of FAK at Ser^910^ was due to ERK5 but not ERK1/2, and we then suggested a role for Ser^910^ in the control of cell motility. In addition, ERK5 had targets in addition to FAK that regulate epithelial-to-mesenchymal transition and cell motility in cancer cells. Taken together, our findings uncover a cancer metastasis-promoting role for ERK5 and provide the rationale for targeting ERK5 as a potential therapeutic approach.

**Electronic supplementary material:**

The online version of this article (10.1007/s13238-020-00701-1) contains supplementary material, which is available to authorized users.

## Introduction

Lung cancer remains the leading cause of cancer-related death worldwide, among which non-small cell lung cancer (NSCLC) accounts for approximately 85% of all types of lung cancer (Siegel et al., 2018). Over the past decade, despite the development of new therapeutic options for cancer treatment, the prognosis of patients with lung cancer still remains unsatisfactory due to the lack of understanding of the molecular mechanisms of intrapulmonary and extrapulmonary metastasis, especially for invasive and metastatic NSCLC. Therefore, exploring the mechanisms underlying the metastasis and invasion of lung cancer is important for establishing new therapeutic targets to improve lung cancer treatment.

Metastasis is a complex process by which tumor cells disseminate from their primary site and form secondary tumors at a distant site. Epithelial-mesenchymal transition (EMT) has been reported to be widely involved in the process of tumorigenicity, tumor invasion and distant metastasis, including in lung cancer, and EMT plays an essential role in the early steps of metastasis (Guarino et al., 2007). Strategies to prevent EMT during malignant progression might have potential as advanced cancer treatments.

Focal adhesion kinase (FAK, also known as protein tyrosine kinase 2, PTK2) is a nonreceptor tyrosine kinase that has essential roles in integrin-induced signal transduction (Schaller, 2010), and it has been found to be overexpressed and activated in the initiation and progression of various malignancies, including head and neck, breast, and ovarian carcinoma (Golubovskaya, 2010; Lee et al., 2015; Yoon et al., 2015). FAK can be phosphorylated on serine, threonine (Ma et al., 2001; Hanks et al., 2003; Grigera et al., 2005) and tyrosine residues (Parsons, 2003). Upon stimulation by multiple growth factor receptors, such as vascular endothelial growth factor receptor, platelet-derived growth factor receptor, and epithelial growth factor receptor, FAK undergoes a conformational change, enabling autophosphorylation of Tyr^397^ at its N-terminal domain (Zhao and Guan, 2011; Brami-Cherrier et al., 2014; Lee et al., 2015), which creates a binding site for Src. The formation of the FAK-Src complex then triggers multiple downstream signaling pathways related to cell proliferation, invasion, migration, and death, which are critical for malignant tumor progression (Parsons, 2003; Cai et al., 2008; Hao et al., 2009; Provenzano and Keely, 2009; Lechertier and Hodivala-Dilke, 2012; Lee et al., 2015; Kleinschmidt and Schlaepfer, 2017). Several serine residues are located near the protein-protein interaction sites of the FAK-Src complex; for example, the proline-rich domain mediates binding the SH3 domain of CAS and the FAT domain mediates binding of paxillin, indicating their participation in protein binding and downstream signaling. Previous studies have found that the CDK (cyclin-dependent kinase) or MAPK (mitogen-activated protein kinase) families could potentially phosphorylate some of the serine residues. It has been reported that Ser^910^ could be phosphorylated by ERK (extracellular regulated kinases) 1/2 in cells stimulated by various agonists, such as phorbol ester PDB (phorbol 12,13-dibutyrate) and growth factors (Hunger-Glaser et al., 2003; Hunger-Glaser et al., 2004). However, another study reported that Ser^910^ of FAK is phosphorylated by ERK5 (also known as mitogen-activated protein kinase 7, MAPK7) but not ERK1/2 under both basal conditions and PMA stimulation (Villa-Moruzzi, 2007). These results suggest that the link between FAK Ser^910^ phosphorylation and the MAPK family and its involvement in tumor progression are still not fully understood, and further studies are warranted to delineate the functional contribution of FAK Ser^910^ to carcinogenesis.

Interestingly, the MAPK signaling pathway has been reported to be widely dysregulated in many types of cancer. There are four groups of conventional MAPKs: c-Jun N-terminal kinase (JNK), ERK1/2, p38, and ERK5. These MAPKs play important roles in many cellular activities, such as regulating gene expression, apoptosis, immune responses, differentiation and mitosis. Among these, ERK5 is the least-studied member of the mammalian MAP kinase cascade, which has been demonstrated to be related to the survival, growth, and differentiation of cancer cells. Recently, aberrant ERK5 signaling has been found in various malignancies, such as glioma, breast cancer, colorectal cancer, renal cancer, prostate cancer and lung cancer (Wu et al., 2016; Zhuang et al., 2016; Liu et al., 2017; Salinas-Sanchez et al., 2017; Won et al., 2017), and it is generally associated with poorer prognosis. When stimulated by growth factors, oxidative stress, and high osmotic pressure, MEK5 may double phosphorylate the TRY motif of ERK5, leading to increase in its activity (Mody et al., 2003). Importantly, ERK5 has also been reported to be critical for prostate cancer metastasis (Mehta et al., 2003) and for angiogenesis in lung carcinoma and melanoma xenografts (Hayashi et al., 2005). In different types of cancer, the active role of the MEK5-ERK5 pathway in supporting cell migration as well as local and distant invasion has been described. For example, in meningiomas IOMM-Lee cells, ERK5 may be involved in HER-2-mediated migration and invasion (Wang et al., 2016); in OVCAR-3 ovarian cancer cells, ERK5 promotes the expression of type II collagen, thereby promoting tumor cell invasion and migration (Dai et al., 2015); in Huh-7 and HepG2 HCC cells, ERK5 mediates EGF or hypoxia-driven migration and invasion (Rovida et al., 2015). In addition, in HeLa cells, ERK5 is required for EGF-induced invasion *in vitro*, in which ERK5 is involved in the MEF2D/DDIAS (DNA damage-induced apoptosis inhibitory factor)/β-catenin pathway (Im et al., 2016). These findings indicate that ERK5 signaling activation might be associated with several human cancers and poor disease survival, which makes it a desirable potential target for cancer therapy. However, the definite regulation and function of ERK5 signaling in cancer metastasis is still poorly understood and remains to be elucidated in detail, which is of great significance for understanding the development of tumors and provides promising directions for cancer treatment.

Herein, we used a high-content proteomics screen in combination with Metacore^TM^ GeneGo pathway analysis to identify ERK5 as an important effector of cancer metastasis and invasion, which is regulated by the phosphorylation of FAK at Ser^910^ and by TGF-β-mediated EMT in lung cancer cells.

## Results

### ERK5 is highly expressed in human lung cancer

**Figure 1:**
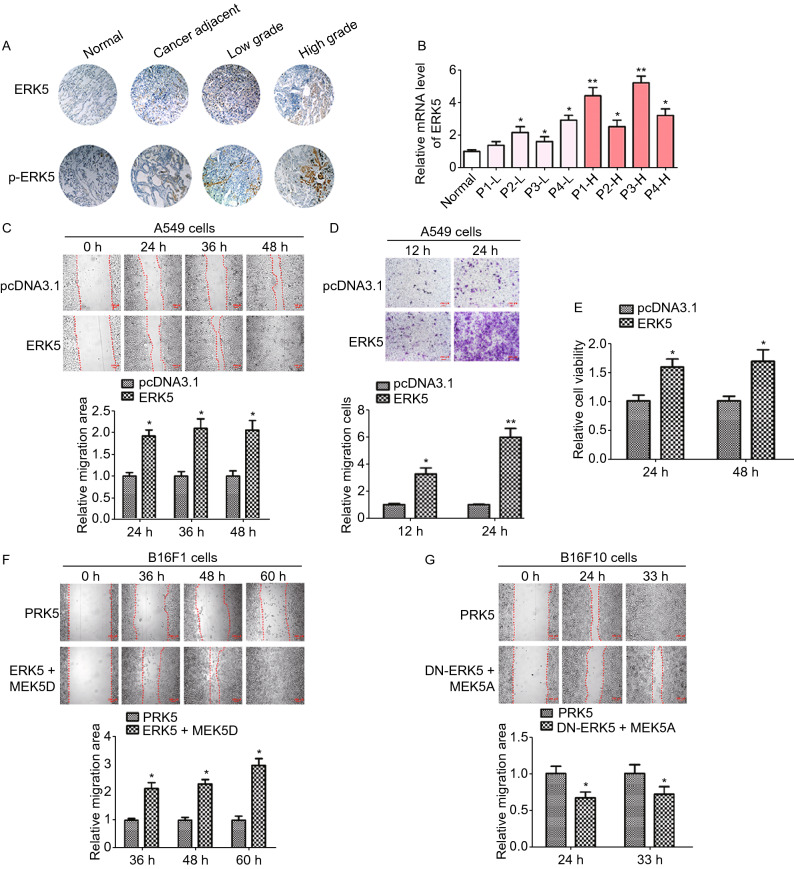
**Overexpression or activation of ERK5 promotes migration of human lung cancer A549 cells and mouse melanoma cells**. (A and B) Expression of ERK5 and p-ERK5 in lung cancer tissues and adjacent specimens. (A) Representative results of ERK5 and p-ERK5 staining micrographs of lung tissues (×20) analyzed by IHC. (B) The mRNA level of ERK5 was measured by RT-qPCR in human lung cancer sections. P1-L-P4-L indicated patient 1 to patient 4 with low grade. P1-H-P4-H indicated patient 1 to patient 4 with high grade. Data are represented as mean ± SD. (C) Wound healing assay was performed on ERK5-overexpression A549 cells (A549-ERK5). Relative scratch covered area was quantified by Image J from four areas. (D) Representative images of transwell-based cell invasion of ERK5-overexpression A549 cells. Matrigel was coated on the bottom of the well as the basement membrane matrix for invasion assay. The invasion cell numbers were quantified by Image J in 12 random fields from three independent experiments. (E) Relative cell viability of ERK5-overexpression A549 cells were measured by MTT assay. (F) Wound healing assay was performed on B16F1 cells transfected with MEK5D and ERK5 or control vector. Relative scratch covered area was quantified by Image J from four areas. (G) Wound healing assay was performed on B16F10 cells transfected with MEK5A and DN-ERK5 or control vector. Relative scratch covered area was quantified by Image J from four areas. Data are represented as mean ± SD, *P < 0.05 and **P < 0.01 compared with respective control or indicated treatment

To evaluate ERK5 involvement in lung cancer, we first examined ERK5 expression in human lung cancer specimens of different grades by immunohistochemistry staining and quantitative reverse transcription PCR (RT-qPCR). The results showed that the levels of ERK5 and phosphorylated ERK5 were consistently higher in lung cancer tissues than those in normal lung tissues. In addition, phosphorylation levels of ERK5 were much higher in high-grade lung cancer tissues than those in low-grade lung cancer tissues, indicating that ERK5 activation correlated with lung cancer malignancy (Fig. 1A). Consistently, the mRNA level of ERK5 in human lung cancer specimens was also elevated (Fig. 1B). In addition, Kaplan-Meier survival analysis of LUAD (lung adenocarcinoma) patients also showed that cases with higher expression of ERK5 exhibited poorer overall survival (Supplementary file 2: Fig. S1). These data suggest that ERK5 is highly expressed in lung cancer and may positively correlate with a malignant phenotype.

### Overexpression of ERK5 promotes migration and invasion of cancer cells

We previously generated constructs allowing the expression of ERK5, and introduced the constructs conferring G418 resistance to A549 lung cancer cells. To elucidate whether ERK5 promotes migration and invasion in lung cancer cells, wound healing and transwell invasion assays were performed. Compared with control cells, A549 cells overexpressing ERK5 showed significantly quicker closure of the wound scratch (Fig. 1C) and faster invasion through the Matrigel (Fig. 1D). Additionally, cell viability was elevated following overexpression of ERK5 (Fig. 1E).

To further investigate whether ERK5 promotes migration and invasion in other types of tumor cells, we employed a constitutively active mutant of an upstream kinase of ERK5 named MEK5 (MEK5D), and we expressed it with ERK5 to study functional responses to ERK5 activation in two murine melanoma cell lines (B16F10 and B16F1) with the same origin and genetic background but with different metastatic potency. Wound-healing assays using B16F1 cells coexpressing MEK5D and ERK5 showed more rapid healing than what was observed in the control cells (Fig. 1F). On the other hand, constitutively inactive mutants of ERK5 (DN-ERK5) and MEK5 (MEK5A) were also constructed. We found that 33 h after making a scratch, B16F10 cells migrated into and completely covered the original wound area, whereas those cotransfected with DN-ERK5 and MEK5A failed to cover a substantial portion of the wound (Fig. 1G). In addition, A549 and B16F1 cells transfected with siERK5 interference fragment displayed slower healing as compared to those transfected with siCTRL interference fragment (Supplementary file 2: Figs. S2 and S3). Taken together, these results indicate that the activation of ERK5 was also critical for the migration of A549, B16F10 and B16F1 cells.

### ERK5 is critical for the regulation of cytoskeletal rearrangement

**Figure 2:**
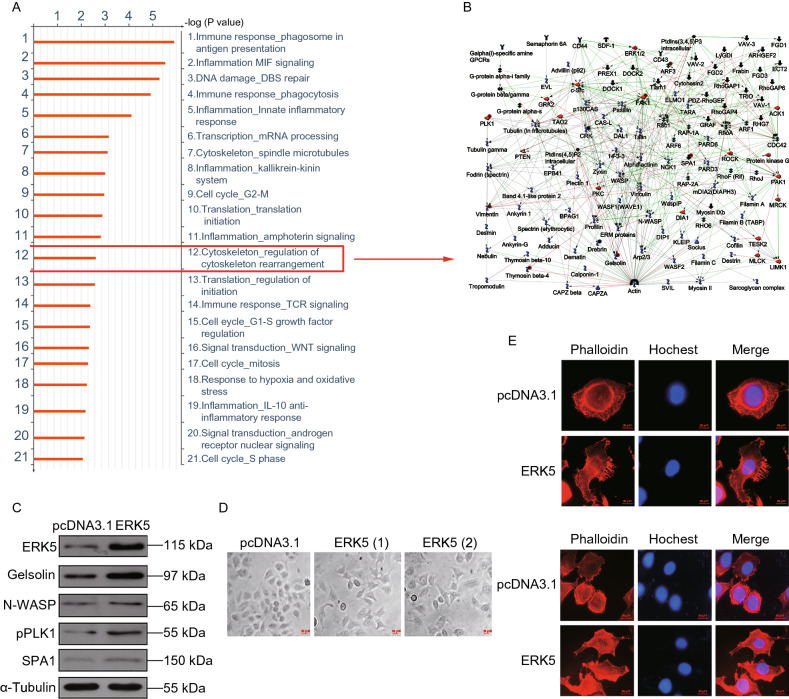
**ERK5 regulates the polymerization of F-actin**. (A) Ontological categories of differentially expressed proteins in ERK5-overexpression A549 cells by Metacore^TM^ GeneGo Pathway Maps analysis. (B) Protein networks associated with the actin regulators differentially expressed in ERK5-overexpression A549 cells (higher definition image is shown in Supplementary file 2: Fig. S4). The network was generated by a shortest paths algorithm of MetaCore^TM^ (GeneGo) software using the list of differentially expressed actin regulators identified by proteomics analysis. (C) Western blot analysis of actin nucleation and polymerization correlated proteins in ERK5-overexpression A549 cells, including ERK5, Gelsolin, N-WASP, p-PLK1and SPA1. (D) The cell shapes of A549 cells overexpressing ERK5 under light microscope. (E) ERK5 regulated the assembly of F-actin. Immunofluorescence was carried out to display F-actin (phalloidin, red), and nuclei (Hochest, blue) in ERK5-overexpression A549 cells and control A549 cells. (F) After serum starvation overnight, the cells were stimulated again with 20% fetal calf serum for 2 h. Then immunofluorescence assay was performed as that described in (E)

To further explore the specific role of ERK5 in cell function and to identify which signaling pathway ERK5 might be involved with, we performed a high-throughput proteomic approach to compare protein expression between A549 and ERK5-A549 cell lines. A *t* test identified 89 differentially expressed proteins (above 2-fold) (Supplementary file 1). The MetaCore^TM^ pathway mapping tool clustered actin regulators from the DEG results (Fig. 2A, 2B and Supplementary file 2: Fig. S4). The protein levels of Gelsolin, N-WASP, p-PLK1, and SPA1 were all increased in ERK5-A549 cell lines (Fig. 2C and Supplementary file 2: Fig. S5). We therefore established that ERK5 was closely related to cytoskeletal rearrangement. Cells migrate by altering their shape and stiffness, leading to a polarized and elongated phenotype (Lauffenburger and Horwitz, 1996). On this basis, we next tested whether ERK5 overexpression alters the morphological changes that are required for cell migration. We found that ERK5-overexpressing cells were more elongated and polarized in shape and exhibited more membrane ruffling at the edge of their cell protrusions (Fig. 2D). Additionally, we observed up to five protrusions in ERK5-overexpressing cells, highlighting their dynamic movement. In contrast, the control cells appeared flatter in shape and were more tightly adhered to the underlying plate (Fig. 2D). Additionally, cytoskeletal changes were examined by immunofluorescence in A549 cells overexpressing ERK5. Consistently, polymerization of F-actin was dependent on ERK5. Phalloidin labeling showed an obvious increase in fibrous actin in ERK5-A549 cells. Cell morphology was changed from spherical to spindle-shaped after ERK5 overexpression (Fig. 2E). Next, after serum starvation overnight, the cells were stimulated again with 20% fetal calf serum for 2 h. We further examined these cells stained with phalloidin and noticed that the actin cytoskeleton in the cell cortex was significantly reorganized in ERK5-overexpressing cells. Longitudinal peripheral distributions of actin filaments were found in ERK5-overexpressing cells, which was in contrast with the accumulated thicker actin filaments in control cells (Fig. 2F). On the other hand, filopodia in ERK5-overexpressing cells were significantly increased compared with the control. Collectively, these data indicate that ERK5 might promote cell migration by modulating the cytoskeletal machinery that is required for cell motility.

### The phosphorylation level of ERK5 is upregulated in highly metastatic cells

**Figure 3:**
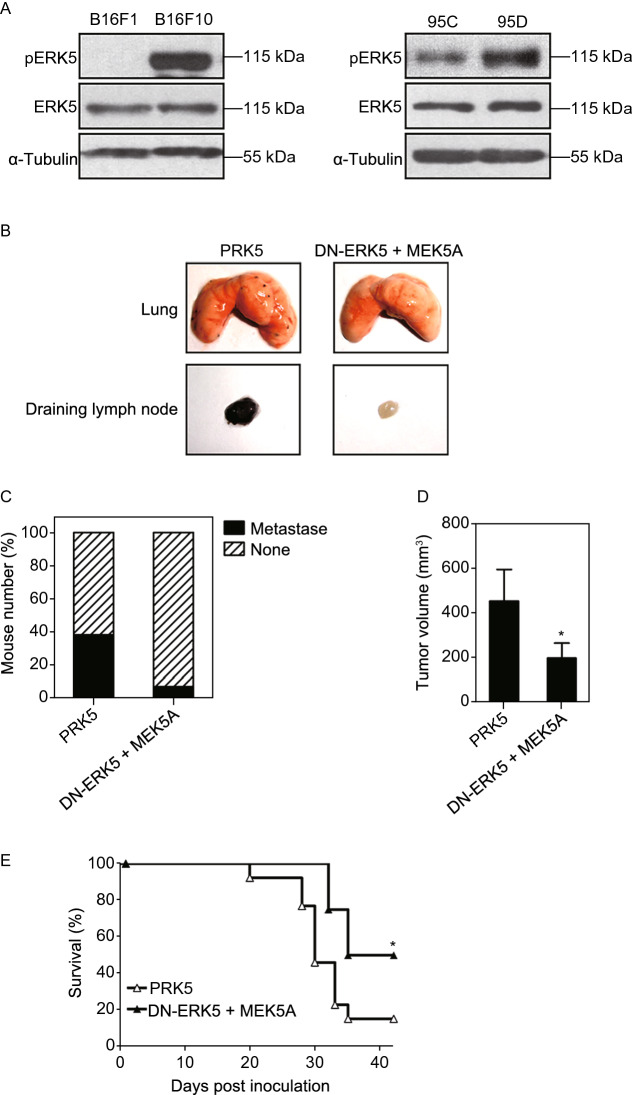
**The phosphorylation level of ERK5 is associated with tumor cell metastasis**. (A) The phosphorylation level of ERK5 in two pairs of cell lines with different metastatic potentials, B16F10 and B16F1, 95D and 95C, as detected by WB analysis. (B) Inactivation of ERK5 inhibited experimental tumor cell metastasis. B16F10 cells transfected with MEK5A and ERK-DN or control vector (5 × 10^4^ cells / mouse) were injected into the left hind footpads of C57BL/6J mice. After 15–20 days, when the tumors formed in situ, intratumoral injection of MEK5A and ERK-DN or control vector was performed every three days. 32 days later, lungs from the mice were resected and analyzed for metastasis. The representative lungs from mice are shown, as well as draining lymph nodes. (C) The metastasis frequency was calculated and Data are presented as mean ± SD of 13 mice. (D and E) Inactivation of ERK5 inhibited the solid tumor growth of the implantable footpad model (D) and prolonged the survival of tumor-bearing mice (E). Data are represented as mean ± SD, *P < 0.05 compared with respective control or indicated treatment

Given that the activation of ERK5 might play an important role in cancer cell migration, we next examined the phosphorylation level of ERK5 in highly metastatic B16F10 and poorly metastatic B16F1 cells. We observed that ERK5 was more highly phosphorylated in B16F10 cells than it was in B16F1 cells (Fig. 3A and Supplementary file 2: Fig. S6). Meanwhile, human giant lung cancer cells (95C, 95D) with low and highly metastatic potential, respectively, were also assessed to measure the association of ERK5 activation with migration ability. We also found that the phosphorylation level of ERK5 was obviously enhanced in highly metastatic 95D cells (Fig. 3A, right panel). These data further confirmed that ERK5 activation was associated with cancer cell metastasis.

The above *in vitro* studies convinced us that inhibition of ERK5 could lead to the suppression of tumor cell invasiveness. Whether the inhibition of ERK5 function in established tumor tissue also can achieve therapeutic efficiency *in vivo* remained an unresolved question. Therefore, efforts were further undertaken to examine the effect of ERK5 on metastasis *in vivo* (Supplementary file 2: Fig. S7A). As expected, multiple black metastatic foci were observed on the lung surfaces of animals injected with the control plasmid PRK5, whereas no obvious tumor nodules were identified in DN-ERK5- and MEK5A-injected animals (Fig. 3B). In lymph nodes (LNs) isolated from tumor-bearing animals, black metastatic foci were found in the control group injected with PRK5; however, lymph nodes from DN-ERK5- and MEK5A-injected animals remained unaffected (Fig. 3B). The frequency of metastasis of B16F10 cells is presented in Fig. 3C; DN-ERK5 and MEK5A coexpression displayed a more potent inhibitory effect on the occurrence of both lymph node and lung metastasis than did the control. In addition, the solid tumor growth in tumor-bearing mice was also inhibited by transfection with DN-ERK5 and MEK5A (Fig. 3D). Furthermore, Kaplan-Meier survival analysis showed that coexpression with DN-ERK5 and MEK5A significantly improved survival, and the 42-day survival rate was 50% and 15% for DN-ERK5 + MEK5A and PRK5, respectively (Fig. 3E). These results show that not only the malignant metastasis of tumors but also primary solid tumor growth was related to the activation of ERK5 in tumor cells.

### ERK5 overexpression upregulates FAK

**Figure 4:**
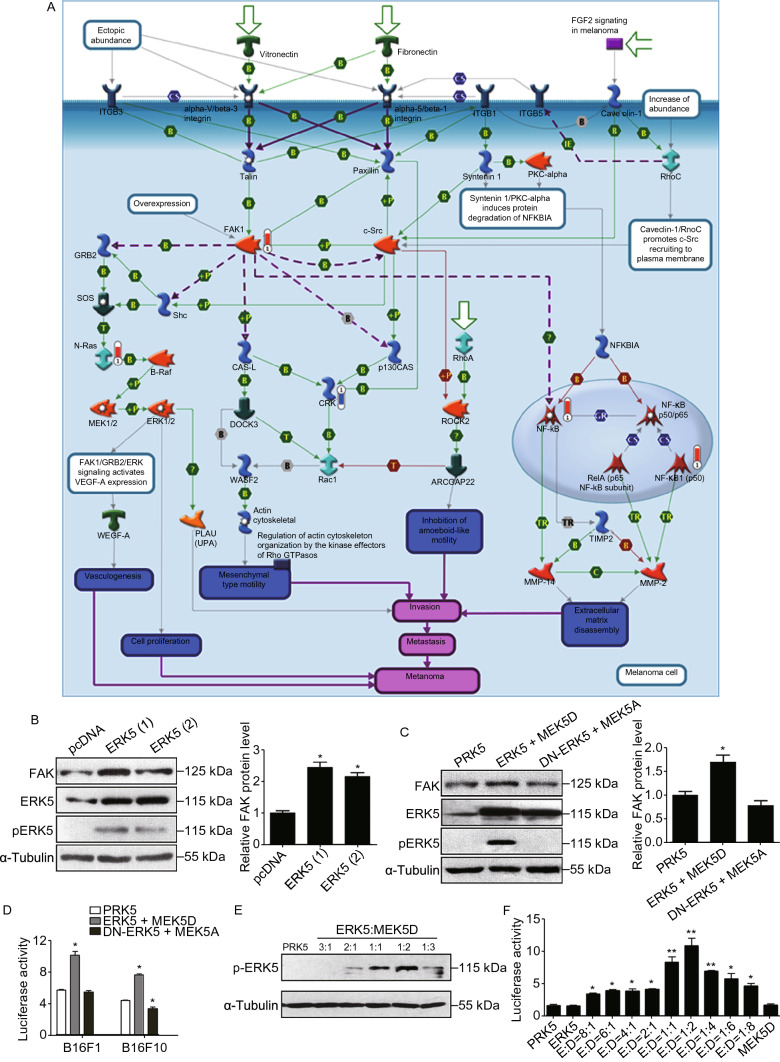
**ERK5 regulates the FAK signaling**. (A) GeneGO pathway showing changes in expression of proteins involved in FAK signaling (higher definition image is shown in Supplementary file 2: Fig. S9). The various proteins on this map are represented by different symbols (representing the functional class of the protein). Thermometers with blue or red shading next to symbols depict proteins identified in the present study: blue color represents the proteins that were downregulated in ERK5-overexpression A549 cells relative to control A549 cells; red color represents the proteins that were upregulated. (B) The expression levels of FAK, ERK5, and p-ERK5 in ERK5-overexpression A549 cells. Band intensity was quantified by Image J software. (C) The expression levels of FAK, ERK5, and p-ERK5 in B16F1 cells transfected with MEK5D + ERK5, MEK5A + DN-ERK5, or control vector PRK5. Band intensity was quantified by Image J software. (D) B16F10 and B16F1 cells were transfected with MEK5D + ERK5, MEK5A + DN-ERK5, or control vector PRK5. Transfected cells were then cotransfected with FAK-Luc plasmid and pRL-TK Renilla vector. After 24 h, luciferase expression was measured. FAK luciferase activity was normalized to that of Renilla and expressed to control. (E and F) The phosphorylation level of ERK5 (E) and the FAK luciferase activity (F) in B16F1 cells transfected with ERK5 and MEK5D at different ratio. Data are represented as mean ± SD, *P < 0.05 and **P < 0.01 compared with respective control or indicated treatment

It has been well characterized that the FAK signaling pathway plays a critical role in tumor metastasis and invasion in various types of tumors. Here, we analyzed the correlation between ERK5 and FAK. We performed MetaCore^TM^ pathway enrichment analysis using proteomics data, and we obtained 50 enrichment pathways from the DEGs (Supplementary file 2: Fig. S8). Among them, FAK1 signaling (*P* = 1.76 × 10^−3^) had a strong positive correlation with ERK5 (Fig. 4A and Supplementary file 2: Fig. S9). We then checked the protein level of FAK in ERK5-overexpressing A549 cells. As shown in Fig. 4B and Supplementary file 2: Fig. S10A, the total FAK level was greatly elevated in response to ERK5 overexpression. Furthermore, to investigate the association of FAK and ERK5 in B16F1 cells, ERK5 and MEK5D or DN-ERK5 and MEK5A overexpression vectors were introduced into the cells. We found that the activation of ERK5 elevated the protein level of FAK, whereas the inactivation of ERK5 decreased the expression of FAK (Fig. 4C and Supplementary file 2: Fig. S10B). Dual luciferase reporter assays demonstrated that the activation of ERK5 significantly stimulated the transcriptional activity from the FAK promoter in both B16F1 and B16F10 cells, while inactivation of ERK5 resulted in the inhibition of FAK only in highly metastatic B16F10 cells (Fig. 4D). Next, we transfected B16F1 cells with ERK5 and MEK5D at different ratios and found that the phosphorylation level of ERK5 increased in a dose dependent manner with increasing amounts of MERK5D; ERK5:MEK5D at a ratio of 1:2 led to the peak phosphorylation value (Fig. 4E). Consistently, the luciferase reporter assays also showed that transcriptional activity of the FAK promoter reached a peak value when the ratio of ERK5 to MEK5D was 1:2 (Fig. 4F). These data suggest that the transcriptional activation of FAK is closely associated with the phosphorylation level of ERK5.

### Identification of USF1 as a major trans-acting factor involved in the ERK5-mediated regulation of FAK gene transcription

**Figure 5:**
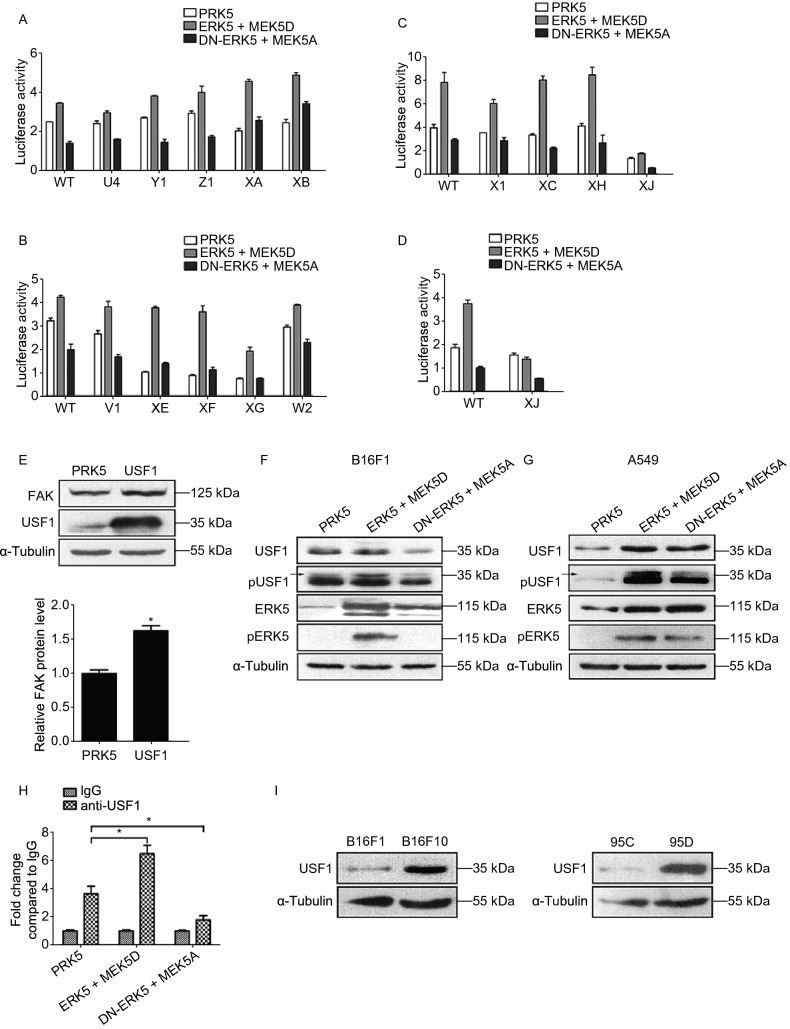
**USF1 is identified as a major trans-acting factor involved in the ERK5-mediated regulation of FAK gene transcription**. (A–C) Transcription activity of the mutations of FAK promoter from dual-luciferase assay with deletion of different NFκB binding sites (A), PEA3, Pax3, or GATA1 binding sites (B), or different USF binding sites (C). (D) Transcription activity of the FAK promoter of XJ mutant. (E) The expression level of FAK in USF1-overexpression B16F1 cells. (F and G) The expression levels of USF1, p-USF1, ERK5, and p-ERK5 in B16F1 cells (F) or A549 cells (G) transfected with MEK5D + ERK5, MEK5A + DN-ERK5, or control vector PRK5. (H) ChIP-qPCR assays were performed to determine the binding of USF1 to FAK promoter regions in B16F1 cells transfected with MEK5D + ERK5, MEK5A + DN-ERK5, or control vector PRK5. (I) The expression level of USF1 in two pair of cell lines with different metastatic potentials, B16F10 and B16F1, 95D and 95C, as detected by WB analysis. Data are represented as mean ± SD, *P < 0.05

Our lab has previously reported that nucleotides from −170 to +43 constitute the minimal mouse FAK promoter that is responsible for basal FAK transcription (Chen et al., 2018). After P-Match analysis, which is a new method for identifying transcription factor (TF) binding sites in DNA sequences, we found that the core promoter region of FAK might contain four USF binding sites, three NFκB/Rel binding sites, two cEts-1/PEA3 binding sites, one GATA1 binding site and one Pax-3 binding site (Supplementary file 2: Fig. S11A). To identify the specific transcription factors involved in ERK5-mediated regulation of FAK gene transcription, we have previously generated a series of deletion constructs carrying a promoter driving expression of a Luc reporter gene (Supplementary file 2: Fig. S11B). First, we focused on the mutant constructs with deletion of different NFκB binding sites, U4, Y1, Z1, XA, and XB. The distinct patterns of promoter activities stimulated by ERK5 in different states were similar to the wild-type (WT) control, indicating that the regulation of FAK by ERK5 was not mediated by NFκB (Fig. 5A). Similarly, there were no apparent changes in mutant constructs with deletions of PEA3, Pax3, or GATA1 binding sites (V1, XE, XF, XG, and W2), suggesting that these TFs were also not responsible for the regulation of FAK by ERK5 (Fig. 5B). However, when we studied the mutant constructs with deletion of different USF binding sites, we found that the effects of ERK5 on the mutants X1, XC and XH were similar to that of the WT, while the mutant XJ displayed a pattern that was different from the control (Fig. 5C). We observed that overexpression of ERK5 and MEK5D failed to stimulate the FAK promoter activity of the XJ mutant, which suggested that the −40 USF binding site might be critical for the regulation of FAK (Fig. 5D).

To examine whether the increased FAK transcription was a result of enhanced USF1 binding to the promoter region, we constructed a USF1 overexpression plasmid. Western blot analysis demonstrated that overexpression of USF1 in B16F1 cells resulted in an increase in FAK protein levels (Fig. 5E). We further investigated whether ERK5 had an effect on the regulation of USF1. In B16F1 cells, coexpression of ERK5 and MEK5D increased the phosphorylation level of USF1 (Fig. 5F). Similar results were also found in A549 cells; both USF1 and its phosphorylation level were markedly elevated in cells transfected with ERK5 and MEK5D (Fig. 5G). In addition, to further confirm whether FAK was a direct target of USF1, we performed a ChIP assay in B16F1 cells, and DNA fragments bound by endogenous USF1 were immunoprecipitated by an anti-USF1 antibody. Normal mouse IgG serum was used as a negative control. Quantitative PCR analysis revealed that USF1 could bind to FAK promoter regions, and the binding was enhanced in B16F1 cells transfected with ERK5 and MEK5D; however, while in those cells transfected with DN-ERK5 and MEK5A, the binding was reduced (Fig. 5H). These data suggest that sustained activation of ERK5 regulated USF1 phosphorylation and its protein stability and enhanced the direct binding of USF1 to the promoter of FAK.

The above results raised the possibility that B16F10 cells might have higher USF1 expression levels than B16F1 cells. This hypothesis was next confirmed by Western blot analysis (Fig. 5I, left and Supplementary file 2: Fig. S12A). In addition, higher USF1 expression levels were also observed in highly metastatic 95D cells than they were in 95C cells (Fig. 5I, right and Supplementary file 2: Fig. S12B), which is consistent with the increased phosphorylation level of ERK5 and the elevation of FAK protein levels in highly metastatic cells, as shown above. Moreover, according to the online cancer transcriptome database Oncomine, USF1 and FAK were found to be overexpressed in both lung adenocarcinoma and lung squamous cell carcinoma patients (Supplementary file 2: Fig. S13). These data suggest that ERK5 might upregulate the expression of FAK through the transcription factor USF1.

### Phosphorylation of FAK Ser^910^ plays a critical role in metastasis

**Figure 6:**
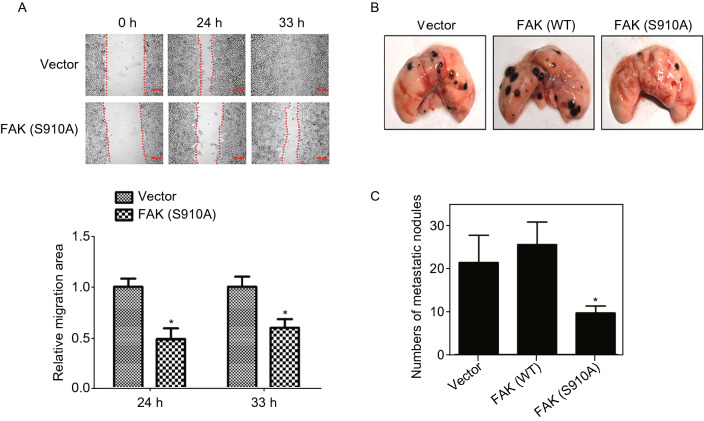
**Phosphorylation of FAK Ser**^**910**^ **is essential for cancer cell metastasis**. (A) Wound healing assay was performed on B16F10 cells transfected with FAK(S910A) and control plasmid. (B) Phosphorylation of FAK Ser^910^ inhibited experimental tumor cell metastasis. The method for establishment of mice model is shown in Supplementary file 2: Fig. S7B. 32 days later, lungs from the mice were resected and analyzed for metastasis and the representative lungs from mice are shown. (C) After fixation in Bouin’s solution, metastatic nodules found on lung surface are counted and averaged. Data are represented as mean ± SD, *P < 0.05 compared with respective control

FAK can be phosphorylated on tyrosine, serine and threonine residues. The phosphorylation tyrosine residues of FAK has been well studied; however, less is known about phosphorylation of serine residues. Nevertheless, Ser^910^ was reported to be phosphorylated by ERK5 under both basal conditions and after PMA stimulation (Villa-Moruzzi, 2007). Thus, the phosphorylation status of FAK Ser^910^ was also analyzed in our study. Wild-type (WT) FAK and a nonphosphorylated FAK (S910A) mutant were then constructed to examine the functional significance of FAK Ser^910^ phosphorylation. Using a wound healing assay, we found that FAK (S910A) overexpression significantly slowed the rate of cells in the wounded area compared to that of the control group at 24 and 33 h (Fig. 6A). To determine whether the antimetastatic effect of FAK (S910A) in B16F10 melanoma cells can also be reproduced *in vivo*, we generated a tail vein metastasis model (Supplementary file 2: Fig. S7B). Intravenous injection of wild-type control B16F10 cells or B16F10 cells transfected with FAK-WT into mice resulted in apparent lung metastasis, but there was no significant difference between the groups. However, when B16F10 cells transfected with FAK (S910A) were intravenously injected into mice, a significant reduction in lung metastatic nodules was observed (Fig. 6B and 6C).

### Effect of ERK5 on the phosphorylation of FAK Ser^910^

**Figure 7:**
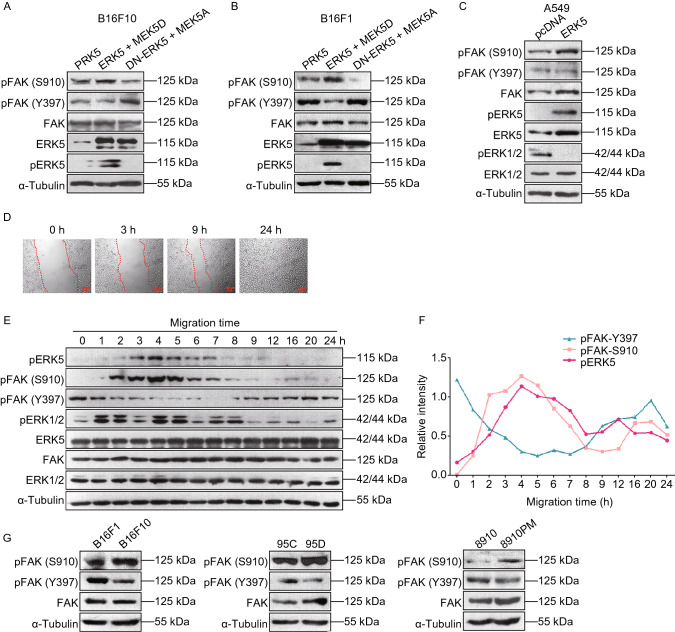
**Regulation of ERK5 on the phosphorylation of FAK Ser**^**910**^. (A and B) WB analysis was performed to detect the expression levels of p-FAK (S910), p-FAK (Y397), FAK, ERK5, and p-ERK5 in B16F10 cells (A) or B16F1 cells (B) transfected with MEK5D + ERK5, MEK5A + DN-ERK5, or control vector PRK5 using specific antibodies. (C) The expression levels of p-FAK (S910), p-FAK (Y397), FAK, ERK5, p-ERK5, ERK1/2, and p-ERK1/2 in ERK5-overexpression A549 cells. (D) Wound healing assay was performed on NIH3T3 cells. The image of cells at different time points are shown. (E) The expression levels of p-FAK (S910), p-FAK (Y397), FAK, ERK5, p-ERK5, ERK1/2, and p-ERK1/2 in NIH3T3 cells at indicated time points during migration. (F) Band intensity of p-FAK (S910), p-FAK (Y397) and p-ERK5 at different time points quantified by Image J software is shown. (G) The expression levels of p-FAK (S910), p-FAK (Y397), and FAK in three pairs of cell lines with different metastatic potentials, B16F10 and B16F1, 95D and 95C, 8910 and 8910PM, as detected by WB analysis

Since the phosphorylation of FAK Ser^910^ is critical for the metastasis of B16F10 cells, we next wanted to clarify the role of ERK5 on FAK Ser^910^ phosphorylation. We found that in B16F10 and B16F1 cells, coexpression of ERK5 and MEK5D resulted in enhanced FAK pSer^910^ levels, which were markedly decreased in cells cotransfected with DN-ERK5 and MEK5A (Fig. 7A and Supplementary file 2: Fig. S14, Fig. 7B and Supplementary file 2: Fig. S15). In addition, while the pSer^910^ level was upregulated in response to the activation of ERK5, phosphorylation at the Tyr^397^ autophosphorylation site was downregulated; this was more apparent in B16F1 cells (Fig. 7A and 7B). Similarly, upon ERK5 interference or treatment with ERK5 selective inhibitor XMD8-92, which targets ERK5’s ATP site to achieve specific inhibition of ERK5 (Umapathy et al., 2014; Wright et al., 2020), the protein level of FAK pSer^910^ decreased significantly, while the expression level of pTyr^397^ increased significantly (Supplementary file 2: Fig. S16 and Fig. S17). On the other hand, in A549 cells stably transfected with ERK5, the pSer^910^ level of FAK was also enhanced, while the pTyr^397^ level was decreased compared to that of the control cells (Fig. 7C and Supplementary file 2: Fig. S18). Meanwhile, we noticed that the phosphorylation of ERK1/2 was inhibited when ERK5 was activated (Fig. 7C). These data indicate that only activation of ERK5 could lead to the phosphorylation of FAK at the Ser^910^ site. Taken together, our results suggest that whether in mouse melanoma B16F10 and B16F1 cells or in human lung cancer A549 cells, the activation of ERK5 could result in the phosphorylation of FAK at Ser^910^, but the phosphorylation at Tyr^397^ is suppressed.

### The levels of pERK5 and pFAK (S910) change synchronously during cell migration

To further characterize the specific role of pERK5 in cell migration, we utilized a wound healing assay to examine the expression levels of pERK5 and pFAK (S910) during cell migration. The mouse fibroblast cell line NIH3T3 was first used to observe the dynamic process of cell migration. We found that 3 h after making a scratch, the closest cells moved towards the wound as sheets or groups, indicating a polarized morphology. Wound closure occurred approximately 24 h after making the scratch (Fig. 7D). Considering the process of wound closure, we selected 0, 1, 2, 3, 4, 5, 6, 7, 8, 9, 12, 16, 20, and 24 h as time points for scratch analysis. As shown in Fig. 7E, while the total protein amounts of ERK5 and FAK remain unchanged, the levels of pERK5 and pFAK (S910) increased and were at a maximum at 4 h after making the scratch, but the levels then decreased steadily to the baseline level at 24 h. The tendency of pERK5 and pFAK (S910) levels to change during cell migration was synchronous. In contrast, the level of pFAK (Y397) decreased and was at a minimum at 7 h after the scratch was made, but then levels were slowly restored to the starting level at 24 h. Meanwhile, we noticed that the total protein level of ERK1/2 displayed no obvious change. However, the phosphorylation level of ERK1/2 fluctuated during the process of migration; it reached its peak value at 1 and 4 h and finally decreased to a low level at 24 h after the scratch was made (Fig. 7E). These data suggested that in the process of NIH3T3 cell migration, the different phosphorylation sites of FAK were alternatively activated; in the early stage, the level of pFAK (S910) was upregulated, and the level of pFAK (Y397) was downregulated; while in the late stage, the level of pFAK (S910) was downregulated, and the level of pFAK (Y397) was upregulated, which worked together to coordinate events during cell migration. Importantly, in this whole process, the levels of pERK5 and pFAK (S910) always changed synchronously (Fig. 7F).

### The phosphorylation level of pFAK (S910) is increased in highly metastatic tumor cells

Since there was a dynamic change in the levels of pFAK (S910) and pFAK (Y397) during NIH3T3 cell migration, we next wanted to evaluate the phosphorylation status of FAK Ser^910^ and FAK Tyr^397^ in highly metastatic tumor cells. Three pairs of cell lines with different metastatic potentials (B16F10 and B16F1, 95D and 95C, and 8910PM and 8910) were used to examine the expression levels of pFAK (S910) and pFAK (Y397). We observed that in highly metastatic cells (B16F10, 95D, and 8910PM), the total protein level of FAK was slightly elevated; however, the level of pFAK (S910) was obviously increased, while that of pFAK (Y397) was significantly decreased (Fig. 7G and Supplementary file 2: Fig. S19). We observed higher expression of ERK5 and pFAK (S910) and lower expression of pFAK (Y397) in highly metastatic cells than we did in cells with low metastatic ability, which indicated that their activity and regulation might be critical for tumor metastasis.

### ERK5 has targets in addition to FAK in the regulation of EMT in lung cancer cells

**Figure 8:**
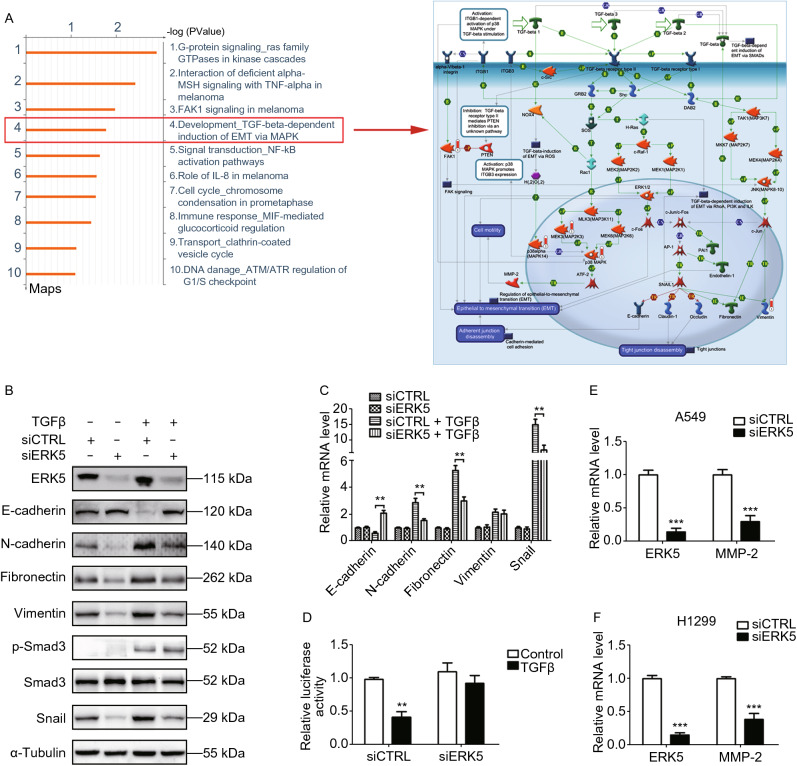
**ERK5 has more targets other than FAK to regulate EMT in lung cancer cells**. (A) GeneGO pathway showing changes in expression of proteins involved in EMT process. The various proteins on the right map (higher definition image is shown in Supplementary file 2: Fig. S20) are represented by different symbols (representing the functional class of the protein). Thermometers with blue or red shading next to symbols depict proteins identified in the present study: blue color represents the proteins that were downregulated in ERK5-overexpression A549 cells relative to control A549 cells; red color represents the proteins that were upregulated. (B) Knockdown of ERK5 altered the expression of EMT-related genes in A549 cells treated with or without TGF-β1, which was examined by western blotting analysis. (C) Quantitative RT-PCR analysis of epithelial and mesenchymal markers. A549 cells were treated as described in (B). (D) A549 cells co-transfected with E-cadherin promoter reporter plasmid (E-cadherin-Luc) and control siRNA (siCTRL) or ERK5 siRNA (siERK5) were incubated with or without TGF-β1 (5 ng/mL) for 24 h. Luciferase activities were normalized on the basis of β-galactosidase expression to adjust for variation in transfection efficiency. (E and F) The mRNA level of MMP2 was obviously reduced upon siERK5 treatment in A549 (E) and H1299 (F) cells. Relative mRNA levels of genes were normalized to β-actin and siCTRL was set as 1.0. Histograms in this figure are shown as means ± SD. **P < 0.01, ***P < 0.001

It is well known that EMT confers migration and invasion properties to cancer cells. Thus, we tested whether ERK5 regulated EMT in lung cancer cells. Indeed, the MetaCore^TM^ pathway mapping tool revealed that five proteins among the DEGs were involved in TGF-β-dependent induction of the EMT pathway: FAK, p38alpha (MAPK14), MEK3, p38 MAPK, and vimentin, as shown in Fig. 8A and Supplementary file 2: Fig. S20. Since loss of E-cadherin is considered to be a fundamental event in EMT, we examined the expression of E-cadherin and its transcription regulators after knocking down ERK5. As expected, the siRNA-mediated ablation of ERK5 in A549 cells completely prevented TGF-β-induced changes in EMT marker expression at both the protein and mRNA levels (Fig. 8B and 8C). Compared to siCTRL-transfected cells, the levels of the epithelial indicator E-cadherin remained high, while the mesenchymal markers N-cadherin, Fibronectin, Vimentin, and Snail did not increase in their expression in the absence of ERK5. The phosphorylation of SMAD3, an essential mediator of TGF-β signaling, was not affected in response to the knocking down of ERK5 (Fig. 8B and Supplementary file 2: Fig. S21). Consistently, ERK5 siRNA restored TGF-β-mediated repression of the E-cadherin promoter (Fig. 8D). Additionally, the mRNA level of MMP2 was decreased in ERK5 knockdown-A549 and H1299 cells as measured by qPCR (Fig. 8E and 8F). We next assessed whether ERK5 contributed to TGF-β-mediated EMT through its enzymatic kinase activity. A549 cells were treated with an ERK5-specific inhibitor, XMD8-92. As was the case with blocking ERK5 by siRNA-mediated gene silencing, treatment with XMD8-92 completely prevented TGF-β-induced EMT of A549 cells, as determined by immunofluorescence staining and by the analysis of E-cadherin expression (Supplementary file 2: Fig. S22). Furthermore, it has been reported previously that USF1 could bind to the promoter region of TGF-β1 (Zhu et al., 2005). Therefore, we speculated that the ERK5-mediated EMT process might be due to its regulation of USF1. To verify our hypothesis, we used a luciferase reporter assay to test whether USF1 could bind to the TGF-β1 promoter and regulate its promoter activity. We showed that coexpression of ERK5 and MEK5D increased the luciferase activity of the reporter containing the TGF-β1 promoter, but it did not affect that of the same reporter in A549 and H1299 cells transfected with USF1 siRNA (Supplementary file 2: Fig. S23). The data indicate that USF1 can bind to the TGF-β1 promoter to increase its transcriptional activity in lung cancer cells. Collectively, these results suggest that knockdown of ERK5 may suppress EMT in lung cancer cells and subsequent metastasis, which is likely to occur because of its regulation of USF1.

## Discussion

The ERK5 pathway exhibits many features that are structurally and functionally distinct from other MAPKs, all of which make it a potentially ideal therapeutic target (Hoang et al., 2017). Recently, an increasing number of reports have indicated that ERK5 signaling might play an important role in cancer progression. Upregulation of ERK5 in many types of cancer, such as human lung cancer and prostate cancer, exhibited an invasive and migratory phenotype, thus contributing to elevated metastasis capacities (Ramsay et al., 2011; Park et al., 2016). Furthermore, pharmacological inhibition of ERK5 suppressed cell migration and invasion in some types of cancer cells *in vitro* (Sawhney et al., 2009; Rovida et al., 2015), and it reduced metastasis of liver and prostate cancer xenografts *in vivo* (Ramsay et al., 2011; Rovida et al., 2015). It has also been reported that ERK5 could regulate tobacco smoke-induced pulmonary, hepatic and urocystic EMT *in vivo* and *in vitro* (Geng et al., 2015; Liang et al., 2015a; Liang et al., 2015b; Liang et al., 2017; Min et al., 2017). Our team previously reported that ERK5 could increase the radioresistance of lung cancer cells by enhancing DNA damage responses (Jiang et al., 2019). Here, we show for the first time that ERK5 might be involved in regulating the expression of FAK and its phosphorylation at Ser^910^, thus contributing to cancer metastasis and invasion.

Aberrant ERK5 signaling is increasingly being linked to a poor disease prognosis and aggressive phenotypes in some types of cancer. Previous studies in prostate cancer revealed that a high level of ERK5 expression was related to metastasis to bone and to a poor prognosis (Mehta et al., 2003). In addition, a high level of p-ERK5 expression was found to be associated with a more advanced tumor stage in oral squamous cells (Sticht et al., 2008). In this study, our immunohistochemical and qPCR analyses show that ERK5 is expressed at different levels in majority of human lung cancer, suggesting its correlation with lung cancer progression. We observed that ERK5 was highly expressed in high-grade lung cancer, and its expression was lower in low-grade lung cancer. These results indicated that ERK5 expression is positively related to more advanced cancer stages. Furthermore, we observed that overexpression of ERK5 or constitutive activation of ERK5 promoted the proliferation, migration and invasion of A549 cells, while constitutive inactivation of ERK5 suppressed the invasive phenotypes of lung cancer cells. In addition, the levels of p-ERK5 were also higher in highly metastatic B16F10 and human giant lung cancer 95D cells than they were in B16F1 and 95C cells, which have poor metastatic ability; these results reveal a link between ERK5 and tumor metastasis. *In vivo* studies also demonstrated that constitutive inactivation of ERK5 reduced lymph node and lung metastasis of B16F10 cells. Thus, our data indicate that ERK5 is indispensable for cancer cell proliferation, migration, and invasion.

Actin polymerization and nucleation are critical processes for actin assembly and cytoskeleton construction, and they have been recognized as the basis for tumor cell spreading, migration, invasion, and metastasis. Metacore^TM^ GeneGo enrichment analysis of differentially expressed proteins revealed that most of the actin regulators were upregulated in ERK5-overexpressing A549 cells, such as gelsolin, N-WASP, p-PLK1, and SPA1, which was further confirmed with subsequent experiments. Overexpression of ERK5 clearly promoted F-actin assembly, caused cell spreading, and subsequently enabled lung cancer cells to move.

Metacore^TM^ GeneGo enrichment analysis of differentially expressed proteins identified the FAK signaling pathway, a complicated pathway involved in cell motility, cell proliferation, and cell survival. We observed that the expression level of FAK was elevated in ERK5-overexpressing A549 cells. Further study demonstrated that ERK5 could regulate FAK expression through the transcription factor upstream stimulatory factor 1 (USF1), which is a member of the basic helix-loop-helix leucine zipper (bHLH-LZ) family. USF1 can regulate the transcription of genes containing enhanced-box (E-box) pyrimidine-rich motifs and initiator elements in their promoter regions. In our study, activation of ERK5 resulted in an increase in the phosphorylation level of USF1 and promoted its binding to the promoter region of the FAK gene. Furthermore, USF1 transcriptionally elevated FAK expression by directly binding to its promoter, thereby promoting cancer metastasis and invasion. In addition, the phosphorylation level of USF1 paralleled ERK5 activity and was found to be enhanced in highly metastatic B16F10 and 95D cancer cells, suggesting the involvement of USF1 in ERK5-regulated FAK expression and subsequent cell metastasis and invasion.

The phosphorylation of FAK at multiple serine and tyrosine sites regulates its activity and binding with other signaling proteins. In addition to the effect of ERK5 on the expression level of FAK, our data showed a specific effect of ERK5 on the phosphorylation of the Ser^910^ site in FAK. Ser^910^ of FAK was previously reported to be a target of ERK1/2 in response to various stimuli, such as EGF (epidermal growth factor), FGF (fibroblast growth factor), PDGF (platelet-derived growth factor), and the phorbol ester PDB (Hunger-Glaser et al., 2003; Hunger-Glaser et al., 2004). However, other studies further revealed that ERK5, but not ERK1/2, contributed to the phosphorylation of Ser^910^ under both basal conditions and with PMA stimulation (Villa-Moruzzi, 2007, 2011). These results seem to be conflicting and debatable, and thus, the link between Ser^910^ and the MAPK family needs to be further explored. Here, our data supported that during the migration process of cancer cells, the phosphorylation level of Ser^910^ was regulated by ERK5 instead of ERK1/2, at least in the cell types investigated. We found that in the cell migration process, ERK5 activity always paralleled Ser^910^ phosphorylation; meanwhile, the phosphorylation of Tyr^397^ displayed the opposite trend, suggesting the positive effect of ERK5 on Ser^910^ phosphorylation and negative effect on Tyr^397^ phosphorylation. These observations also suggest that the phosphorylation of Ser^910^ and Tyr^397^ might coordinately regulate some critical events during cell migration. Interestingly, while ERK5 was activated, ERK1/2 was simultaneously suppressed, indicating their distinct roles in cell migration. Furthermore, we noticed that activation of ERK5 resulted in elevated Ser^910^ phosphorylation levels, while inactivation of ERK5 led to loss of pSer^910^, thus confirming Ser^910^ phosphorylation by ERK5. More importantly, our *in vivo* experiments further indicated that a nonphosphorylatable FAK (S910A) mutation significantly reduced lung metastasis. Altogether, our findings suggest that ERK5 might promote cancer cell metastasis and invasion by elevating FAK expression and phosphorylating FAK at the Ser^910^ site.

On the other hand, Metacore^TM^ GeneGo enrichment analysis of differentially expressed proteins also revealed that the TGF-β-dependent induction of the EMT pathway was activated as a result of ERK5 overexpression. Indeed, we demonstrate that ERK5 is required for TGF-β-induced EMT in A549 and H1299 cell lines. Knockdown of ERK5 by siRNA strongly impaired the changes in gene expression underlying EMT. In fact, our data conflict with a previous study in which ERK5 knockout in A549 cells, as well as in 4T1 cells, decreases the expression levels of ECAD and finally triggers EMT (Chen et al., 2012). However, our findings are consistent with another study in which ERK5 depletion increased the expression level of ECAD and inhibited EMT in both MDA-MB-231 and A549 cells and thus significantly reduced lung metastases (Javaid et al., 2015). Our data together with the latter report agree on the conclusion that ERK5 is critical for inducing EMT and for maintaining the metastatic ability of cancer cells. In addition, it has also been reported that USF1 could bind to the promoter of TGF-β1 (Zhu et al., 2005). Therefore, we speculate that ERK5 might contribute to cancer metastasis by regulating the TGF-β1-mediated EMT process through USF1, which deserves further investigation in detail.

**Figure 9:**
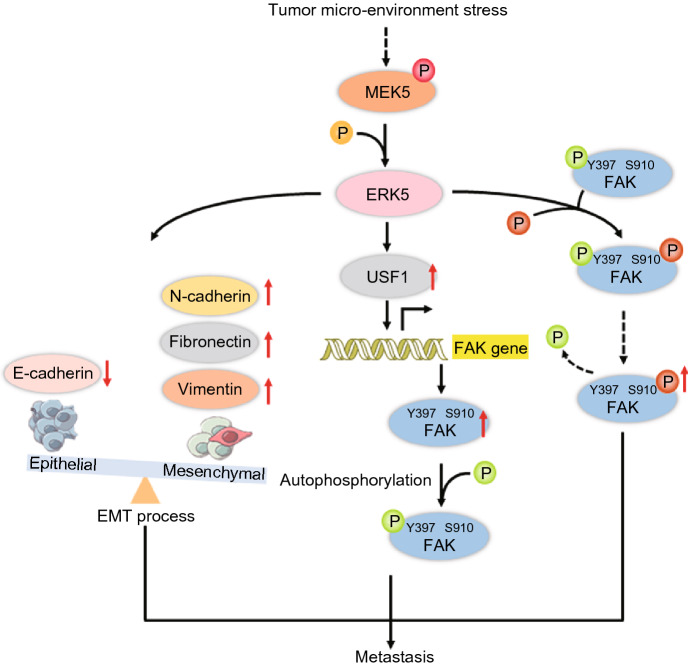
Schematic illustration of the potential mechanisms by which ERK5 promotes cancer cell metastasis

In conclusion, our data demonstrate that ERK5 is highly expressed in lung cancer, directly regulates FAK expression and its phosphorylation at the Ser^910^ site and is required for cell proliferation, F-actin polymerization and epithelial-to-mesenchymal transition, which are associated with cell migration and invasion (Fig. 9). Given the expression of ERK5 in various types of cancer, ERK5 might be a novel target for cancer therapy.

## Materials and Methods

### Materials

Antibodies against ERK5, phospho-ERK5, ERK1/2, phospho-ERK1/2, FAK, N-WASP, Gelsolin, smad3, and p-smad3 were purchased from Cell Signaling Technologies. The antibodies to detect p-PLK1, USF1 were obtained from Abcam (Cambridge, MA, USA). The antibodies against SPA1, E-cadherin, Snail, N-cadherin, Fibronectin, Vimentin, and α-tubulin were from Santa Cruz Biotechnology. Anti-p-USF1 was from ThermoFisher Scientific. The antibodies to detect pSer^910^ and pTyr^397^of FAK were from BioSource (Camarillo, CA, USA). Human ERK5 siRNA, USF1 siRNA, and non-specific control siRNA was commercially obtained from Santa Cruz Biotechnology (USA).

### ERK5, MEK5, USF1 gene clone and variant construction

The sequence of murine ERK5, MEK5 and USF1 was amplified from cDNA of B16F10 cells and cloned into pRK5-Flag empty vector. The double phosphorylation site (Thr218/Tyr220) of ERK5 was inactivated and mutated into a permanent non-phosphorylated form (Ala218/Phe220) (named as DN-ERK5) by the overlap PCR method. Using pRK5-Flag-ERK5 as a template, the upstream fragment was amplified with primer ERK5-c-Upper primer/DNm-lower primer, and the downstream fragment was amplified with primer DNm-upper primer/ERK5-c-Lower primer. The mixture was used as a template, and PCR was performed with primers ERK5-c-Upper primer/ERK5-c-Lower primer. The PCR amplification product was inserted between the BamH I and Xho I digestion sites of the pRK5-Flag vector. MEK5D refers to the permanently phosphorylated activated form (Asp311/Asp315) of MEK5 based on the double phosphorylation site (Ser311/Thr315), and MEK5A refers to the permanent non-phosphorylated form (Ala311/Ala315). The method of preparing MEK5D and MEK5A is the same as that of preparing DN-ERK5. Primers sequences are shown Supplementary file 3: Table S1.

### Cells, cell culture, and transfection

NSCLC cell lines A549 and H1299, human giant-cell lung carcinoma cell lines 95C and 95D, human ovarian cancer cell lines 8910 and 8910PM, mouse melanoma cell lines B16F10 and B16F1, were purchased from the American Type Culture Collection (ATCC, Philadelphia, PA, USA). The stably A549-ERK5 cell line was established previously in our lab (Jiang et al., 2019). These cells were cultured in Dulbecco’s modified Eagle’s medium (DMEM) or RPMI 1640 (Invitrogen, Carlsbad, CA, USA), respectively, supplemented with 10% (*v*/*v*) fetal bovine serum (FBS; Invitrogen, Carlsbad, CA, USA), and 1% penicillin-streptomycin (Invitrogen, Carlsbad, CA, USA). All cells were cultured in a humidified CO_2_ incubator at 37 °C. Plasmids were introduced into cells by Polyethylenimine (PEI)-mediated transfection as described previously (Li et al., 2007). Briefly, cells were seeded at a density of 2 × 10^6^ cells/10 cm dish (Corning, Lowell, USA), and were transfected with PEI-complexed plasmids (at a ratio of 2:1, *w*/*w*) in serum-free DMEM medium. Two hours after addition of the DNA, the medium was switched to fresh complete medium and transfected cells were continuously cultured until harvest for analysis.

### RNA extraction and quantitative real-time PCR

Total RNA was extracted from cells using the TRIzol Kit (Invitrogen), and 1 μg was used for cDNA synthesis primed with Oligo(dT)18 primers (Takara, Dalian, China). Conventional RT-PCRs were carried out with the oligonucleotide primers shown in Supplementary file 3: Table S2. Each RT-PCR reaction was repeated at least 3 times and β-actin was used as an internal control.

### Cell migration assay

The cell migration assay was performed using transwell inserts (8.0 mm pore size, Millipore, Billerica, MA, USA) as described previously (Lin et al., 2013). In brief, the under surface of the membrane was coated with fibronectin (10 µg/mL) in PBS (pH 7.4) at 37 °C for 2 h. The lower chamber was filled with 0.6 mL of 10% FBS supplemented with DMEM medium. Before experiment, cells were serum-starved overnight (DMEM plus 0.5% BSA), then re-suspended in migration medium (DMEM plus 0.5% BSA). 1 × 10^6^ cells in a volume of 0.1 mL were added to the upper chamber. After incubation at 37 °C for 9 h, cells on the upper surface of the membrane were removed. The migrant cells attached to the lower surface were fixed in 10% formalin at room temperature for 30 min, and stained for 20 min with a solution containing 1% crystal violet and 2% ethanol in 100 mmol/L borate buffer (pH 9.0). The number of cells migrating to the lower surface of the membrane was counted in five fields under a microscope with a magnification of ×100. All groups of experiments were conducted in triplicate, and the cell number was counted by Image-Pro Plus 6.0 software.

### Protein extraction, digestion, and labeling with iTRAQ reagents

Cells were harvested when they reached 90% confluence, rinsed three times with ice-cold PBS and collected using cell scrapers after the addition of 200 μL TEAB (0.5 mol/L triethylammonium bicarbonate) dissolution buffer. The samples were broken by the ultrasonic wave for 20 min, and then after centrifugation at 12,000 r/min for 15 min, the supernatant was subsided by adding 4-fold volume of cold acetone containing 10 mmol/L DTT for approximately 2 h. Following centrifugation at 12,000 r/min for 15 min at 4 °C, the pellets were collected and mixed with 800 μL cold acetone at 56 °C to break the proteins’ disulfide bonds. After centrifugation at 12,000 r/min for 15 min at 4 °C, the dried pellets were collected and dissolved with 100 μL TEAB dissolution buffer. The protein concentration was determined by the Bradford protein method.

An aliquot of total protein (100 μg) was dissolved to 100 μL in a dissolution buffer and then diluted with 500 μL 50 mmol/L NH_4_HCO_3_. 2 μg trypsin was added and then incubated overnight at 37 °C. After protein digestion, equal volume of 0.1% formic acid was added for acidification. Peptides were purified on Strata-XC18 pillar, which was first activated with methanol, then balanced by adding 1 mL 0.1% formic acid three times, washed with 0.1% formic acid + 5% acetonitrile twice, and eluted with 1 mL 0.1% formic acid + 80% acetonitrile. The peptides were dried by vacuum centrifugation. The dried peptides powder was redissolved with 20 μL 0.5 mol/L TEAB for peptides labeling.

The peptides were labeled with iTRAQ Reagent-8 plex Multiplex Kit (AB Sciex U.K. Limited) according to the manufacturer’s instructions. The samples and labeled marker were as follows: Samples from A549 cells were labeled with iTRAQ tag 115 and samples from A549-ERK5 cells were labeled with iTRAQ tag 121. All of the labeled samples were mixed with an equal amount. The labeled samples were fractionated using high-performance liquid chromatography (HPLC) system (Thermo DINOEX Ultimate 3000 BioRS) using a Durashell C18 (5 μm, 100 Å, 4.6 × 250 mm).

### LC-MS/MS analysis

Data acquisition was performed with a Triple TOF 5600 System (AB SCIEX, Concord, ON). Samples were chromatographed using a 90 min gradient from 2%–30% (mobile phase A 0.1% (*v*/*v*) formic acid, 5% (*v*/*v*) acetonitrile; mobile phase B 0.1% (*v*/*v*) formic acid, 95% (*v*/*v*) acetonitrile) after direct injection onto a 20 μm PicoFrit emitter (New Objective) packed to 12 cm with Magic C18 AQ 3 μm 120 Å stationary phase. MS1 spectra were collected in the range 350–1,500 m/z for 250 ms. The 20 most intense precursors with charge state 2–5 were selected for fragmentation, and MS2 spectra were collected in the range 50–2,000 m/z for 100 ms; precursor ions were excluded from reselection for 15 s.

### Data analysis

ProteinPilot Software 5.0 (AB SCIEX) was used for identification of proteins and relative iTRAQ quantification. ProteinPilot utilizes Paragon™ database search algorithm (5.0.0.0.4767) and non-linear fitting method to determine the integrated false discovery rate (FDR) for peptide identification and quantification (Shilov et al., 2007; Tang et al., 2008). For FDR calculation, an automatic decoy database search strategy (Choi and Nesvizhskii, 2008) was used to estimate FDR using the PSPEP (Proteomics System Performance Evaluation Pipeline Software) algorithm. Only proteins with at least one unique peptide and unused value more than 1.3 were considered for further analysis. Protein lists were interpreted according to fold change in expression. The cut-off for high abundance (> 1.5-fold over normal, *P* < 0.05) and low abundance (< 0.67-fold over normal, *P* < 0.05) proteins were selected to identify differentially abundant proteins (DAPs) based on biological replicate method.

### Bioinformatics analysis

Differentially expressed proteins identified were further analyzed by pathway analysis using MetaCore^TM^ version 5.4 (GeneGo, St. Joseph, MI). MetaCore^TM^ is a manually curated proprietary database. Gene symbols of dysregulated proteins were uploaded into the database. For enrichment analysis, gene IDs of the uploaded files were matched with gene IDs in GeneGo ontologies in MetaCore^TM^, that included GeneGo Pathway Maps, GeneGo Process Networks, GeneGo Diseases (by Biomarkers), GeneGo Metabolic Networks and GO Processes. For network analysis, shortest paths algorithms were used.

### Tissue microarray assay and immunohistochemistry

To assess ERK5 expression in human lung cancers, a set of lung cancer tissue microarray (TMA) was purchased from Shanghai Outdo Biotech (Hlug-ade050cd-01-T-126, Shanghai, China). The TMA contained 36 lung cancer tissues with matched adjacent non-lung cancer tissues (Table 1). Standard immunohistochemical analysis was used to evaluate ERK5 expression. Briefly, after dried at 62 °C for 30 min, TMA was conventionally dewaxed and hydrated; then cooked with citrate antigen repair solution under high pressure for 1 min. The samples were then treated with 2% H_2_O_2_ for 10 min and blocked with goat serum for 30 min. Rabbit-derived anti-human ERK5 and p-ERK5 antibodies (1:200 dilution, Cell Signaling, USA) was incubated at room temperature for 2 h for immunohistochemical (IHC) detection of the ERK5 protein and its phosphorylation level in TMA samples. After that, TMA was successively incubated with goat anti-rabbit antibodies and ExtrAvidin-conjugated horseradish peroxidase. Staining was developed with the diaminobenzidine (DAB) substrate, and sections were counterstained with hematoxylin. Finally, a light microscopy was used to evaluate the expression of immunohistochemical markers.

**Table 1:** Clinical parameters of lung cancer tissue microarray

**Parameters**	**n (%)**
**Age**	**36**
≤45	
45-60	
≥60	8 (22.22)
15 (41.67)	
13 (36.11)	
**Gender**	**36**
Male	15 (41.67)
Female	21 (58.33)
**Organization type**	**36**
Normal lung tissue	5 (13.89)
Primary lung adenocarcinoma	14 (38.89)
Metastases of lung adenocarcinoma	4 (11.11)
Negative lymph node	7 (19.44)
Positive lymph node	6 (16.67)
**Pathological grade**	**18**
I	3 (16.67)
II	2 (66.67)
III	5 (27.78)
IV	8 (44.44)

### ChIP assays

Briefly, cell extracts were prepared from cross-linked cells that were incubated with anti-USF1 antibody. The immune complexes were collected *via* protein A-agarose and washed thoroughly. The DNA present in the cell extracts and in the immunoprecipitants were purified and quantified by real-time qPCR. The sequences of the PCR primers for the FAK promoter were 5′-CGCACAGCTGGGATACACTTTA-3′ and 5′-TCACCTCAGCGCAGAGCTCTA-3′.

### Wound-healing assay

Cells were plated in 12-well culture plates to form cell monolayer (near 70% confluence). After serum starvation for 12 h, a wound was made with a sterile P-200 micropipette to scrape off the cells. The wells were then washed three times with PBS to remove non-adherent cells. The progress of wound closure was monitored with microphotographs of ×10 magnification taken with light microscope (Carl Zeiss Axioplan 2) at different time points after washing with PBS.

### Western blot analysis

Whole cell lysate was prepared with RIPA buffer (Santa Cruz Biotechnology) containing protease inhibitors, PMSF and orthovanadate. Total protein was denatured by heating and separated on SDS-PAGE gel. After transferring to nitrocellulose membrane and blocking with 5% milk in TBS buffer, the protein of interest was immunoplexed with the indicated primary antibody and corresponding secondary antibody. Bound antibodies were then visualized with ECL plus Western blot detection reagents (GE Healthcare). Signal intensity was quantified by densitometry using the software Image J (NIH, Bethesda, MD). All experiments were done in triplicate and performed at least three times independently.

### Transient transfection and luciferase activity assay

B16F1 or B16F10 cells were seeded at 2 × 10^6^ in a 6-well plate, cultured overnight and transfected using Lipofectamine transfection agent (Invitrogen, USA) according to the manufacturer’s protocol. For normalization of luciferase activity, the pRL-TK control vector encoding Renilla luciferase was used for cotransfection together with pGL3 plasmids. In some experiments, the pGL3-control vector was used in transfection as a positive control of promoter activity, which contains an SV40 promoter plus enhancer sequences resulting in strong expression of luciferase gene in many types of mammalian cells. For all experiments, cells were cultured for 24 h after transfection and lysed with the Passive Lysis Buffer (Promega, USA). Lysates were analyzed using Dual-Luciferase Reporter Assay System kit (Promega, USA). Luminescence was measured on luminometer (Turner Biosystems Instrument, USA). All experiments were performed at least three times.

### Immunofluorescence

Cells were seeded and cultured on glass slides. After 24 h, cells were fixed by 4% paraformaldehyde for 30 min, penetrated by 0.1% Triton X-100 for 15 min, and blocked by 5% BSA for 30 min. To stain the F-actin fibers, cells were incubated for 40 min with Texas Red-X phalloidin. Subsequently, Nuclei were counterstained with DAPI. The images were obtained by inverted ZEISS LSM710 confocal microscope (40× oil lens) (Carl Zeiss), with ZEN 2009 Light Edition software (Carl Zeiss).

### Animals

C57BL/6J mice (6 to 8 weeks of age) were obtained from Shanghai Laboratory of Animal Center (Shanghai, China) and housed in a temperature-controlled sterile room where humidity and light were carefully monitored. Animal welfare and experimental procedures were performed strictly in accordance with high standard animal welfare and other related ethical regulations approved by Nanjing University.

### Mouse melanoma lung metastasis model

To make lung metastasis models, 5 × 10^5^ cells in a volume of 50 μL were injected into the right hind footpads. Tumor volume was monitored by measurement of the two maximum perpendicular tumor diameters with calipers every alternate day. When tumors reached a size of about 5 × 5 mm, the mice were arbitrarily assigned to different groups to receive intratumoral injections of 50 μL M-PEI complexed plasmids. Plasmids (8 μg per injection) were injected for each animal. Injections were repeated every 3 days for a total of 9 days; tumors were measured every other day, and their volumes were calculated. Tumor-bearing mice were killed on the seventeenth day after the first treatment, and lungs and LN were removed for hematoxylin and eosin staining analysis.

### Tail vein metastasis assay

To produce experimental metastasis, the C57BL/6J mice were injected intravenously with 2 × 10^6^ cells in 200 μL of PBS via tail vein. After 15 days, the mice were euthanized, and their lungs were resected and photos were taken (Nikon Coolpix 4500, Japan) before fixation in Bouin’s solution for further analysis. The numbers of metastatic nodules on the surface of the organs were counted macroscopically.

### Statistical analysis

Statistical analysis was carried out using the SPSS software (version 11.0; SPSS, Chicago, IL). Data were expressed as the mean ± standard deviations (SD). For paired data, statistical analyses were performed using two-tailed Student’s* t*-tests. For multiple comparisons, statistical analyses were performed using one-way analysis of variance (ANOVA) with a Tukey post-test. For all analyses, *P* < 0.05 was considered statistically significant.

## Electronic supplementary material

Below is the link to the electronic supplementary material.

Supplementary material 1 (XLSX 28 kb)

Supplementary material 2 (PPTX 17537 kb)

Supplementary material 3 (DOCX 14 kb)

## Abbreviations

CDK: cyclin-dependent kinase
DN: dominant negative
EGF: epidermal growth factor
EMT: epithelial-mesenchymal transition
ERK5: extracellular signal regulated kinase 5
FAK: focal adhesion kinase
FGF: fibroblast growth factor
JNK: Jun N-terminal kinases
MAPKs: mitogen-activated protein kinases
MEK: MAP kinase kinase
NF-κB: nuclear factor κB
NSCLC: non-small cell lung cancer
PDGF: platelet-derived growth factor
TF: transcription factor
TMA: tissue microarray
USF1: upstream stimulatory factor 1

## References

[CR1] Brami-Cherrier K, Gervasi N, Arsenieva D, Walkiewicz K, Boutterin MC, Ortega A, Leonard PG, Seantier B, Gasmi L, Bouceba T (2014). FAK dimerization controls its kinase-dependent functions at focal adhesions. EMBO J.

[CR2] Cai X, Lietha D, Ceccarelli DF, Karginov AV, Rajfur Z, Jacobson K, Hahn KM, Eck MJ, Schaller MD (2008). Spatial and temporal regulation of focal adhesion kinase activity in living cells. Mol Cell Biol.

[CR3] Chen R, Yang Q, Lee JD (2012). BMK1 kinase suppresses epithelial-mesenchymal transition through the Akt/GSK3beta signaling pathway. Cancer Res.

[CR4] Chen X, Li W, Xu C, Wang J, Zhu B, Huang Q, Chen D, Sheng J, Zou Y, Lee YM (2018). Comparative profiling of analog targets: a case study on resveratrol for mouse melanoma metastasis suppression. Theranostics.

[CR5] Choi H, Nesvizhskii AI (2008). False discovery rates and related statistical concepts in mass spectrometry-based proteomics. J Proteome Res.

[CR6] Dai J, Wang T, Wang W, Zhang S, Liao Y, Chen J (2015). Role of MAPK7 in cell proliferation and metastasis in ovarian cancer. Int J Clin Exp Pathol.

[CR7] Geng H, Zhao L, Liang Z, Zhang Z, Xie D, Bi L, Wang Y, Zhang T, Cheng L, Yu D (2015). ERK5 positively regulates cigarette smoke-induced urocystic epithelial-mesenchymal transition in SV40 immortalized human urothelial cells. Oncol Rep.

[CR8] Golubovskaya VM (2010). Focal adhesion kinase as a cancer therapy target. Anticancer Agents Med Chem.

[CR9] Grigera PR, Jeffery ED, Martin KH, Shabanowitz J, Hunt DF, Parsons JT (2005). FAK phosphorylation sites mapped by mass spectrometry. J Cell Sci.

[CR10] Guarino M, Rubino B, Ballabio G (2007). The role of epithelial-mesenchymal transition in cancer pathology. Pathology.

[CR11] Hanks SK, Ryzhova L, Shin NY, Brabek J (2003). Focal adhesion kinase signaling activities and their implications in the control of cell survival and motility. Front Biosci.

[CR12] Hao H, Naomoto Y, Bao X, Watanabe N, Sakurama K, Noma K, Motoki T, Tomono Y, Fukazawa T, Shirakawa Y (2009). Focal adhesion kinase as potential target for cancer therapy (Review). Oncol Rep.

[CR13] Hayashi M, Fearns C, Eliceiri B, Yang Y, Lee JD (2005). Big mitogen-activated protein kinase 1/extracellular signal-regulated kinase 5 signaling pathway is essential for tumor-associated angiogenesis. Cancer Res.

[CR14] Hoang VT, Yan TJ, Cavanaugh JE, Flaherty PT, Beckman BS, Burow ME (2017). Oncogenic signaling of MEK5-ERK5. Cancer Lett.

[CR15] Hunger-Glaser I, Fan RS, Perez-Salazar E, Rozengurt E (2004). PDGF and FGF induce focal adhesion kinase (FAK) phosphorylation at Ser-910: dissociation from Tyr-397 phosphorylation and requirement for ERK activation. J Cell Physiol.

[CR16] Hunger-Glaser I, Salazar EP, Sinnett-Smith J, Rozengurt E (2003). Bombesin, lysophosphatidic acid, and epidermal growth factor rapidly stimulate focal adhesion kinase phosphorylation at Ser-910: requirement for ERK activation. J Biol Chem.

[CR17] Im JY, Yoon SH, Kim BK, Ban HS, Won KJ, Chung KS, Jung KE, Won M (2016). DNA damage induced apoptosis suppressor (DDIAS) is upregulated via ERK5/MEF2B signaling and promotes beta-catenin-mediated invasion. Biochim Biophys Acta.

[CR18] Javaid S, Zhang J, Smolen GA, Yu M, Wittner BS, Singh A, Arora KS, Madden MW, Desai R, Zubrowski MJ (2015). MAPK7 Regulates EMT Features and Modulates the Generation of CTCs. Mol Cancer Res.

[CR19] Jiang W, Jin G, Cai F, Chen X, Cao N, Zhang X, Liu J, Chen F, Wang F, Dong W (2019). Extracellular signal-regulated kinase 5 increases radioresistance of lung cancer cells by enhancing the DNA damage response. Exp Mol Med.

[CR20] Kleinschmidt EG, Schlaepfer DD (2017). Focal adhesion kinase signaling in unexpected places. Curr Opin Cell Biol.

[CR21] Lauffenburger DA, Horwitz AF (1996). Cell migration: a physically integrated molecular process. Cell.

[CR22] Lechertier T, Hodivala-Dilke K (2012). Focal adhesion kinase and tumour angiogenesis. J Pathol.

[CR23] Lee BY, Timpson P, Horvath LG, Daly RJ (2015). FAK signaling in human cancer as a target for therapeutics. Pharmacol Ther.

[CR24] Li S, Dong W, Zong Y, Yin W, Jin G, Hu Q, Huang X, Jiang W, Hua ZC (2007). Polyethylenimine-complexed plasmid particles targeting focal adhesion kinase function as melanoma tumor therapeutics. Mol Ther.

[CR25] Liang Z, Wu R, Xie W, Xie C, Wu J, Geng S, Li X, Zhu M, Zhu W, Zhu J (2017). Effects of Curcumin on Tobacco Smoke-induced Hepatic MAPK Pathway Activation and Epithelial-Mesenchymal Transition In Vivo. Phytother Res.

[CR26] Liang Z, Xie W, Wu R, Geng H, Zhao L, Xie C, Li X, Huang C, Zhu J, Zhu M (2015). ERK5 negatively regulates tobacco smoke-induced pulmonary epithelial-mesenchymal transition. Oncotarget.

[CR27] Liang Z, Xie W, Wu R, Geng H, Zhao L, Xie C, Li X, Zhu M, Zhu W, Zhu J (2015). Inhibition of tobacco smoke-induced bladder MAPK activation and epithelial-mesenchymal transition in mice by curcumin. Int J Clin Exp Pathol.

[CR28] Lin Y, Peng N, Li J, Zhuang H, Hua ZC (2013). Herbal compound triptolide synergistically enhanced antitumor activity of amino-terminal fragment of urokinase. Mol Cancer.

[CR29] Liu F, Zhang H, Song H (2017). Upregulation of MEK5 by Stat3 promotes breast cancer cell invasion and metastasis. Oncol Rep.

[CR30] Ma A, Richardson A, Schaefer EM, Parsons JT (2001). Serine phosphorylation of focal adhesion kinase in interphase and mitosis: a possible role in modulating binding to p130(Cas). Mol Biol Cell.

[CR31] Mehta PB, Jenkins BL, McCarthy L, Thilak L, Robson CN, Neal DE, Leung HY (2003). MEK5 overexpression is associated with metastatic prostate cancer, and stimulates proliferation, MMP-9 expression and invasion. Oncogene.

[CR32] Min J, Geng H, Liu Z, Liang Z, Zhang Z, Xie D, Wang Y, Zhang T, Yu D, Zhong C (2017). ERK5 regulates tobacco smokeinduced urocystic epithelialmesenchymal transition in BALB/c mice. Mol Med Rep.

[CR33] Mody N, Campbell DG, Morrice N, Peggie M, Cohen P (2003). An analysis of the phosphorylation and activation of extracellular-signal-regulated protein kinase 5 (ERK5) by mitogen-activated protein kinase kinase 5 (MKK5) in vitro. Biochemical Journal.

[CR34] Park SJ, Choi YS, Lee S, Lee YJ, Hong S, Han S, Kim BC (2016). BIX02189 inhibits TGF-beta1-induced lung cancer cell metastasis by directly targeting TGF-beta type I receptor. Cancer Lett.

[CR35] Parsons JT (2003). Focal adhesion kinase: the first ten years. J Cell Sci.

[CR36] Provenzano PP, Keely PJ (2009). The role of focal adhesion kinase in tumor initiation and progression. Cell Adh Migr.

[CR37] Ramsay AK, McCracken SR, Soofi M, Fleming J, Yu AX, Ahmad I, Morland R, Machesky L, Nixon C, Edwards DR (2011). ERK5 signalling in prostate cancer promotes an invasive phenotype. Br J Cancer.

[CR38] Rovida E, Di Maira G, Tusa I, Cannito S, Paternostro C, Navari N, Vivoli E, Deng X, Gray NS, Esparis-Ogando A (2015). The mitogen-activated protein kinase ERK5 regulates the development and growth of hepatocellular carcinoma. Gut.

[CR39] Salinas-Sanchez AS, Serrano-Oviedo L, Nam-Cha SY, Roche-Losada O, Sanchez-Prieto R, Gimenez-Bachs JM (2017). Prognostic Value of the VHL, HIF-1alpha, and VEGF Signaling Pathway and Associated MAPK (ERK1/2 and ERK5) Pathways in Clear-Cell Renal Cell Carcinoma. A Long-Term Study. Clin Genitourin Cancer.

[CR40] Sawhney RS, Liu W, Brattain MG (2009). A novel role of ERK5 in integrin-mediated cell adhesion and motility in cancer cells via Fak signaling. J Cell Physiol.

[CR41] Schaller MD (2010). Cellular functions of FAK kinases: insight into molecular mechanisms and novel functions. J Cell Sci.

[CR42] Shilov IV, Seymour SL, Patel AA, Loboda A, Tang WH, Keating SP, Hunter CL, Nuwaysir LM, Schaeffer DA (2007). The Paragon Algorithm, a next generation search engine that uses sequence temperature values and feature probabilities to identify peptides from tandem mass spectra. Mol Cell Proteomics.

[CR43] Siegel RL, Miller KD, Jemal A (2018). Cancer statistics, 2018. CA Cancer J Clin.

[CR44] Sticht C, Freier K, Knopfle K, Flechtenmacher C, Pungs S, Hofele C, Hahn M, Joos S, Lichter P (2008). Activation of MAP kinase signaling through ERK5 but not ERK1 expression is associated with lymph node metastases in oral squamous cell carcinoma (OSCC). Neoplasia.

[CR45] Tang WH, Shilov IV, Seymour SL (2008). Nonlinear fitting method for determining local false discovery rates from decoy database searches. J Proteome Res.

[CR46] Umapathy G, El Wakil A, Witek B, Chesler L, Danielson L, Deng X, Gray NS, Johansson M, Kvarnbrink S, Ruuth K (2014). The kinase ALK stimulates the kinase ERK5 to promote the expression of the oncogene MYCN in neuroblastoma. Sci Signal.

[CR47] Villa-Moruzzi E (2007). Targeting of FAK Ser910 by ERK5 and PP1delta in non-stimulated and phorbol ester-stimulated cells. Biochem J.

[CR48] Villa-Moruzzi E (2011). Tyrosine phosphatases in the HER2-directed motility of ovarian cancer cells: Involvement of PTPN12, ERK5 and FAK. Anal Cell Pathol (Amst).

[CR49] Wang ZY, Wang WJ, Xu S, Wang SS, Tu Y, Xiong YF, Mei JH, Wang CL (2016). The role of MAPK signaling pathway in the Her-2-positive meningiomas. Oncology Reports.

[CR50] Won KJ, Im JY, Kim BK, Ban HS, Jung YJ, Jung KE, Won M (2017). Stability of the cancer target DDIAS is regulated by the CHIP/HSP70 pathway in lung cancer cells. Cell Death Dis.

[CR51] Wright TD, Raybuck C, Bhatt A, Monlish D, Chakrabarty S, Wendekier K, Gartland N, Gupta M, Burow ME, Flaherty PT (2020). Pharmacological inhibition of the MEK5/ERK5 and PI3K/Akt signaling pathways synergistically reduces viability in triple-negative breast cancer. J Cell Biochem.

[CR52] Wu J, Cui H, Zhu Z, Wang L (2016). MicroRNA-200b-3p suppresses epithelial-mesenchymal transition and inhibits tumor growth of glioma through down-regulation of ERK5. Biochem Biophys Res Commun.

[CR53] Yoon H, Dehart JP, Murphy JM, Lim ST (2015). Understanding the roles of FAK in cancer: inhibitors, genetic models, and new insights. J Histochem Cytochem.

[CR54] Zhao X, Guan JL (2011). Focal adhesion kinase and its signaling pathways in cell migration and angiogenesis. Adv Drug Deliv Rev.

[CR55] Zhu Y, Casado M, Vaulont S, Sharma K (2005). Role of upstream stimulatory factors in regulation of renal transforming growth factor-beta1. Diabetes.

[CR56] Zhuang K, Zhang J, Xiong M, Wang X, Luo X, Han L, Meng Y, Zhang Y, Liao W, Liu S (2016). CDK5 functions as a tumor promoter in human colorectal cancer via modulating the ERK5-AP-1 axis. Cell Death Dis.

