# Supplementary material for: Extracellular signal regulated kinase 5 promotes cell migration, invasion and lung metastasis in a FAK-dependent manner
Source: Protein Cell. 2020 Mar 6;11(11):825–45. doi: 10.1007/s13238-020-00701-1 (PMC7647985; doi:10.1007/s13238-020-00701-1)
Supplement: Supplementary file 2 — Supplementary material 2 (PPTX 17537 kb) [file 13238_2020_701_MOESM2_ESM.pptx]

## Slide 1
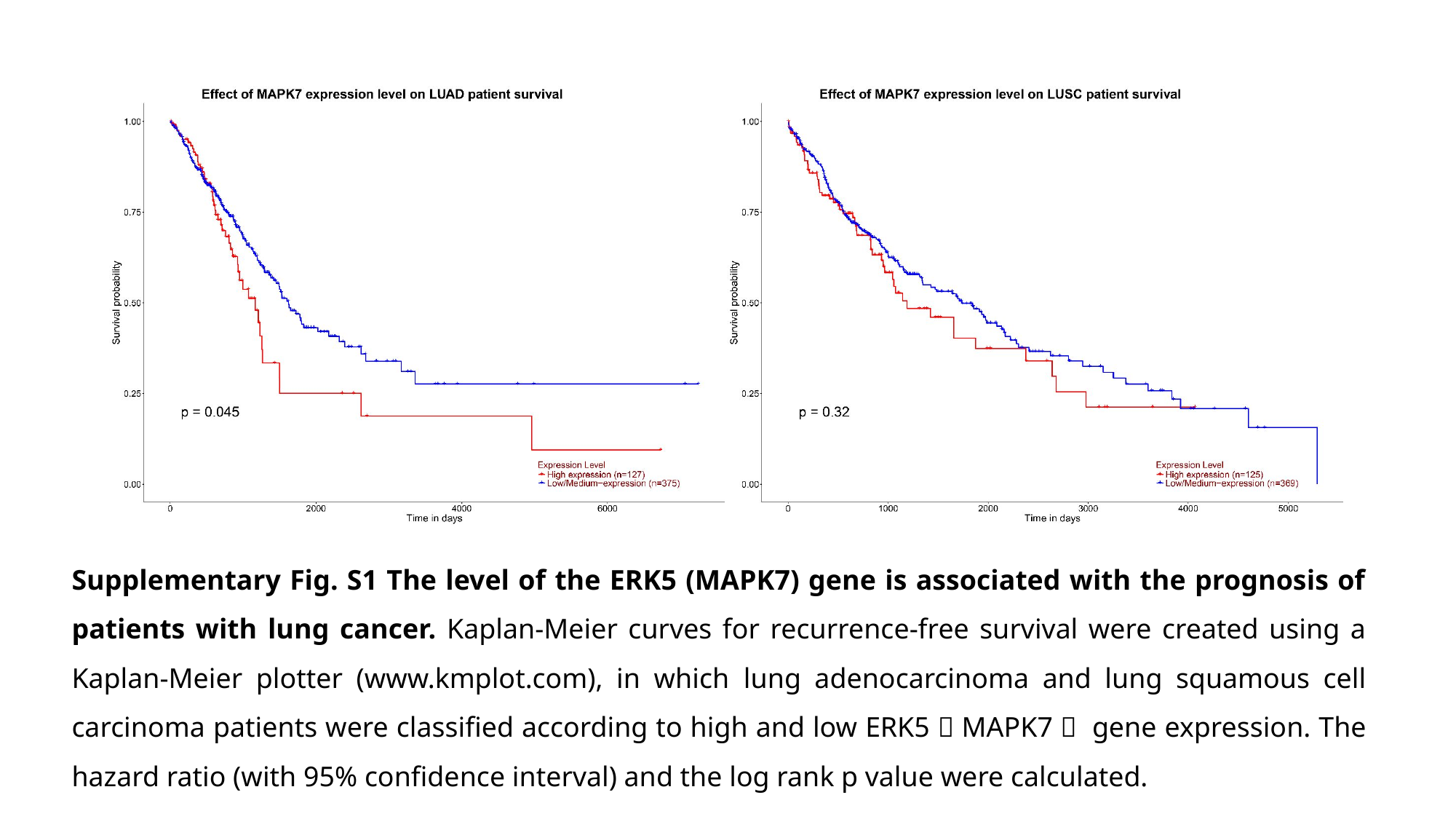

Supplementary Fig. S1 The level of the ERK5 (MAPK7) gene is associated with the prognosis of patients with lung cancer. Kaplan-Meier curves for recurrence-free survival were created using a Kaplan-Meier plotter (www.kmplot.com), in which lung adenocarcinoma and lung squamous cell carcinoma patients were classified according to high and low ERK5（MAPK7） gene expression. The hazard ratio (with 95% confidence interval) and the log rank p value were calculated.

## Slide 2
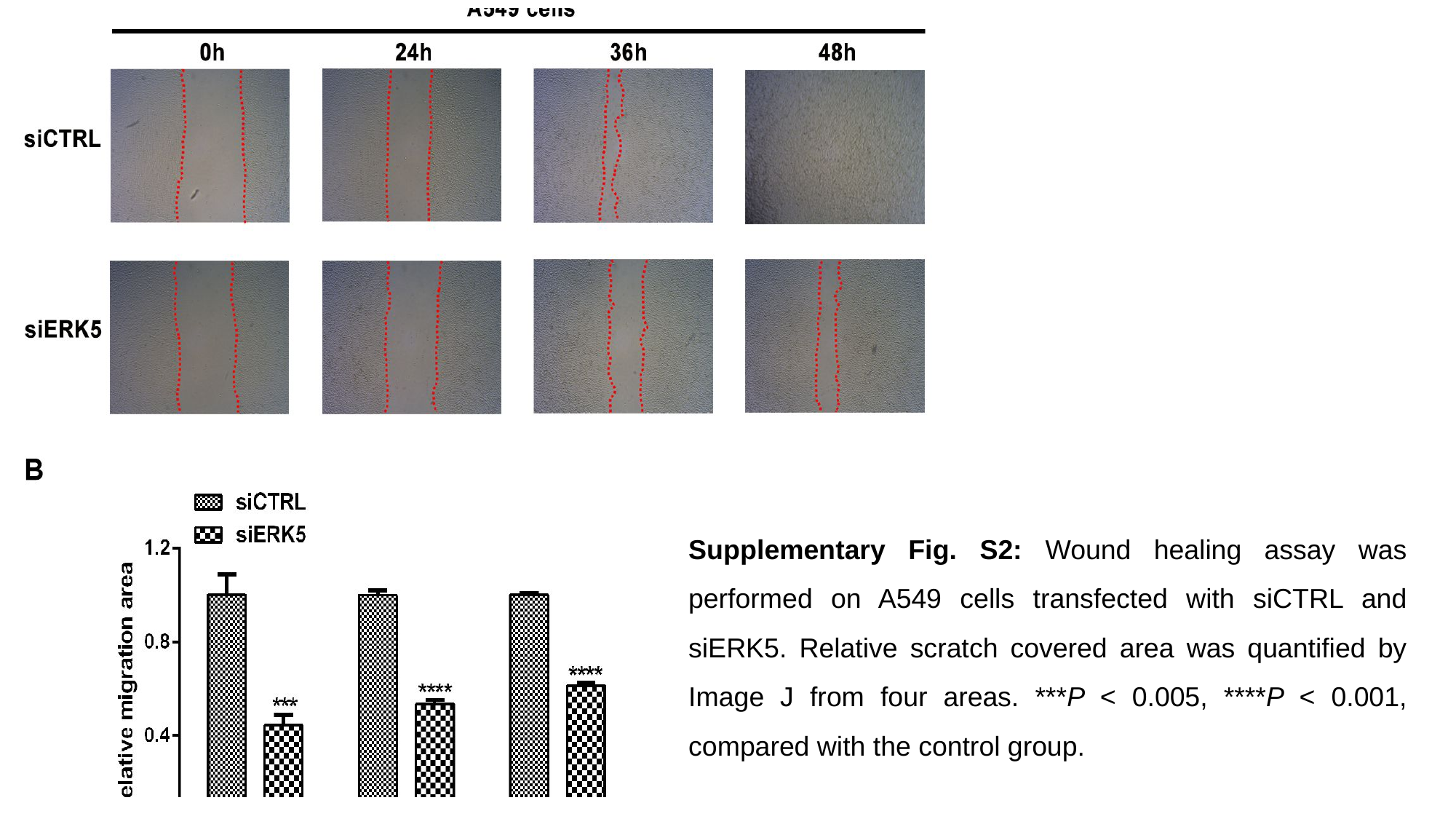

Supplementary Fig. S2: Wound healing assay was performed on A549 cells transfected with siCTRL and siERK5. Relative scratch covered area was quantified by Image J from four areas. ***P < 0.005, ****P < 0.001, compared with the control group.

## Slide 3
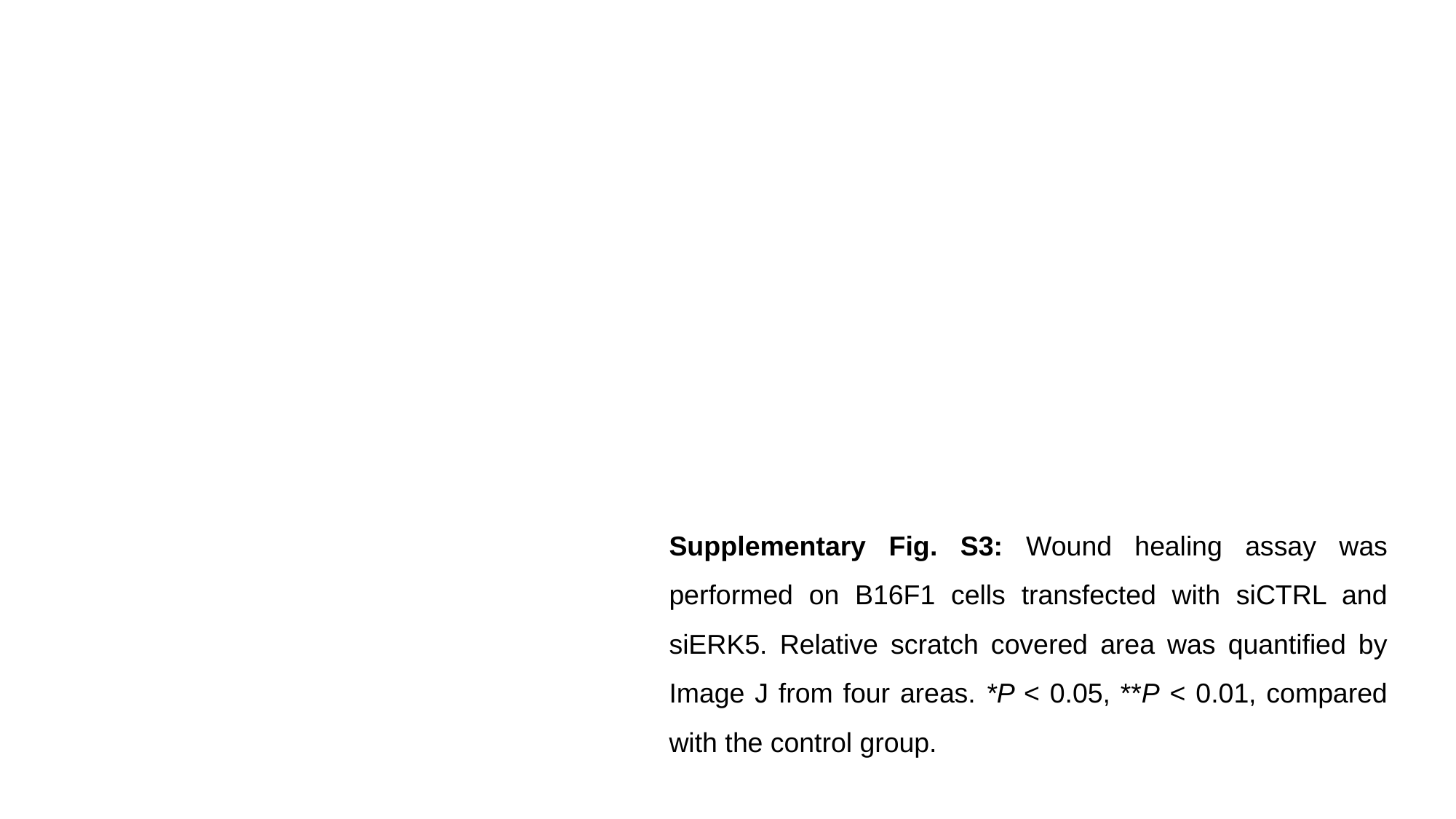

Supplementary Fig. S3: Wound healing assay was performed on B16F1 cells transfected with siCTRL and siERK5. Relative scratch covered area was quantified by Image J from four areas. *P < 0.05, **P < 0.01, compared with the control group.

## Slide 4
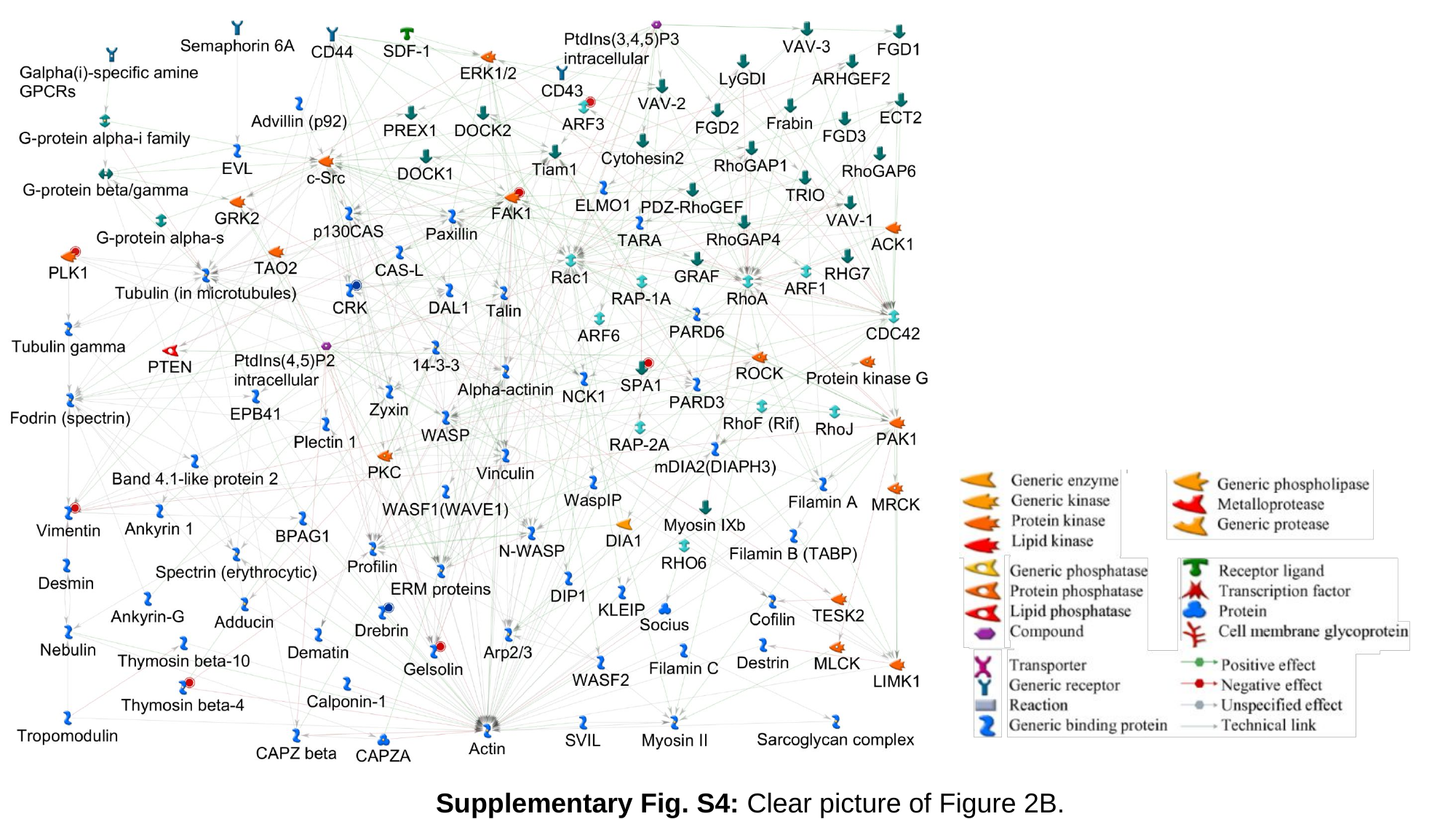

Supplementary Fig. S4: Clear picture of Figure 2B.

## Slide 5
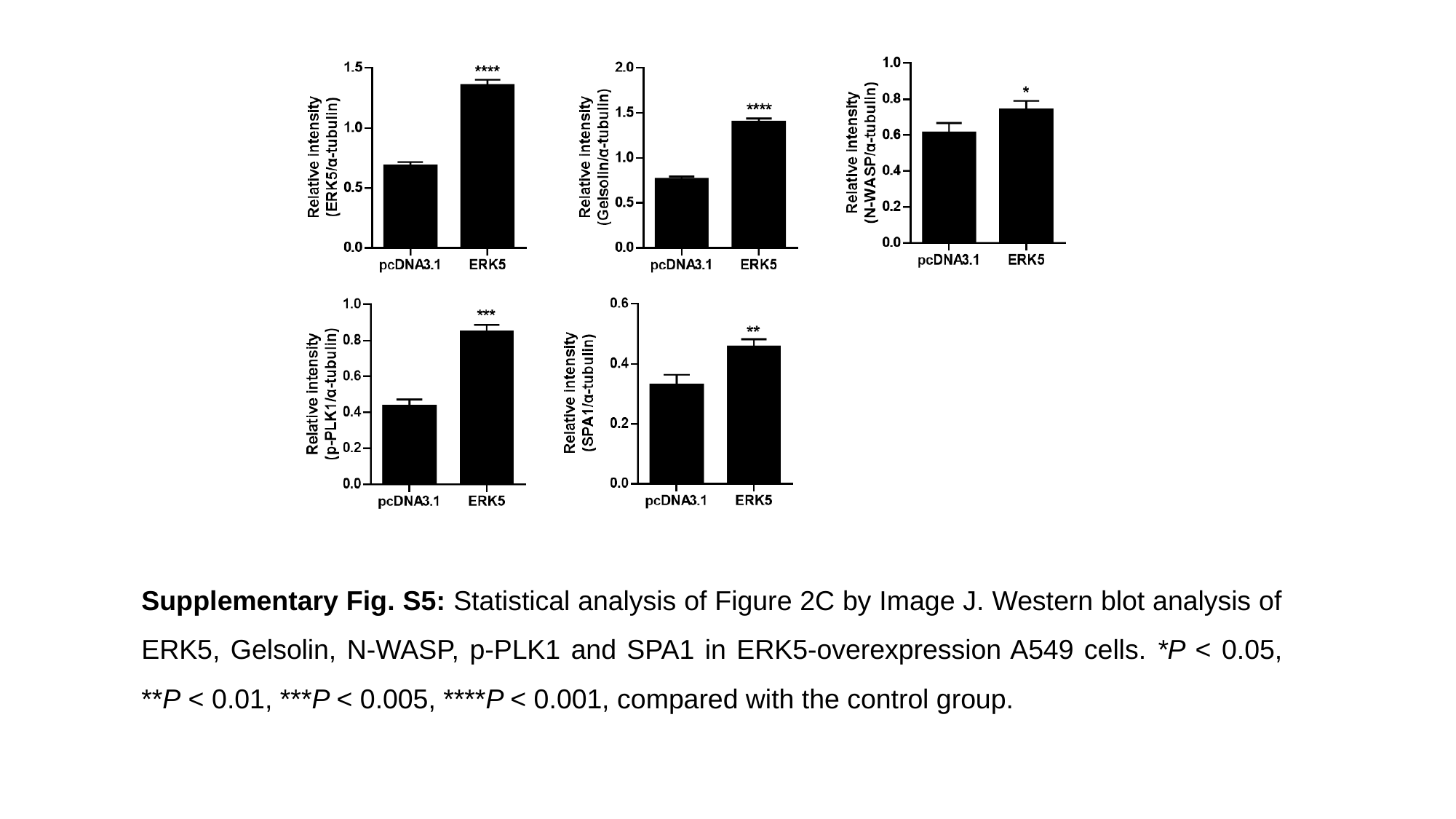

Supplementary Fig. S5: Statistical analysis of Figure 2C by Image J. Western blot analysis of ERK5, Gelsolin, N-WASP, p-PLK1 and SPA1 in ERK5-overexpression A549 cells. *P < 0.05, **P < 0.01, ***P < 0.005, ****P < 0.001, compared with the control group.

## Slide 6
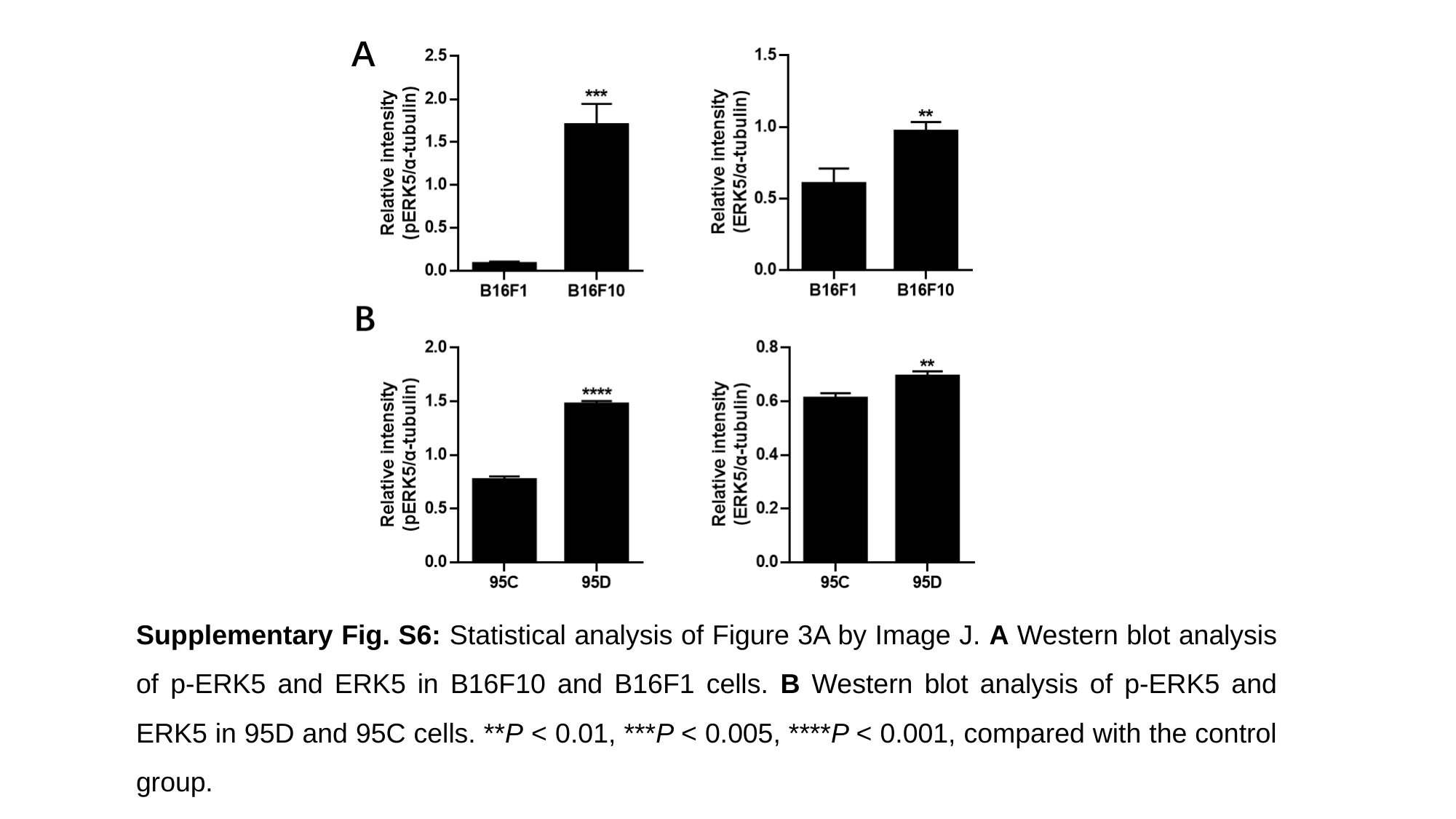

Supplementary Fig. S6: Statistical analysis of Figure 3A by Image J. A Western blot analysis of p-ERK5 and ERK5 in B16F10 and B16F1 cells. B Western blot analysis of p-ERK5 and ERK5 in 95D and 95C cells. **P < 0.01, ***P < 0.005, ****P < 0.001, compared with the control group.

## Slide 7
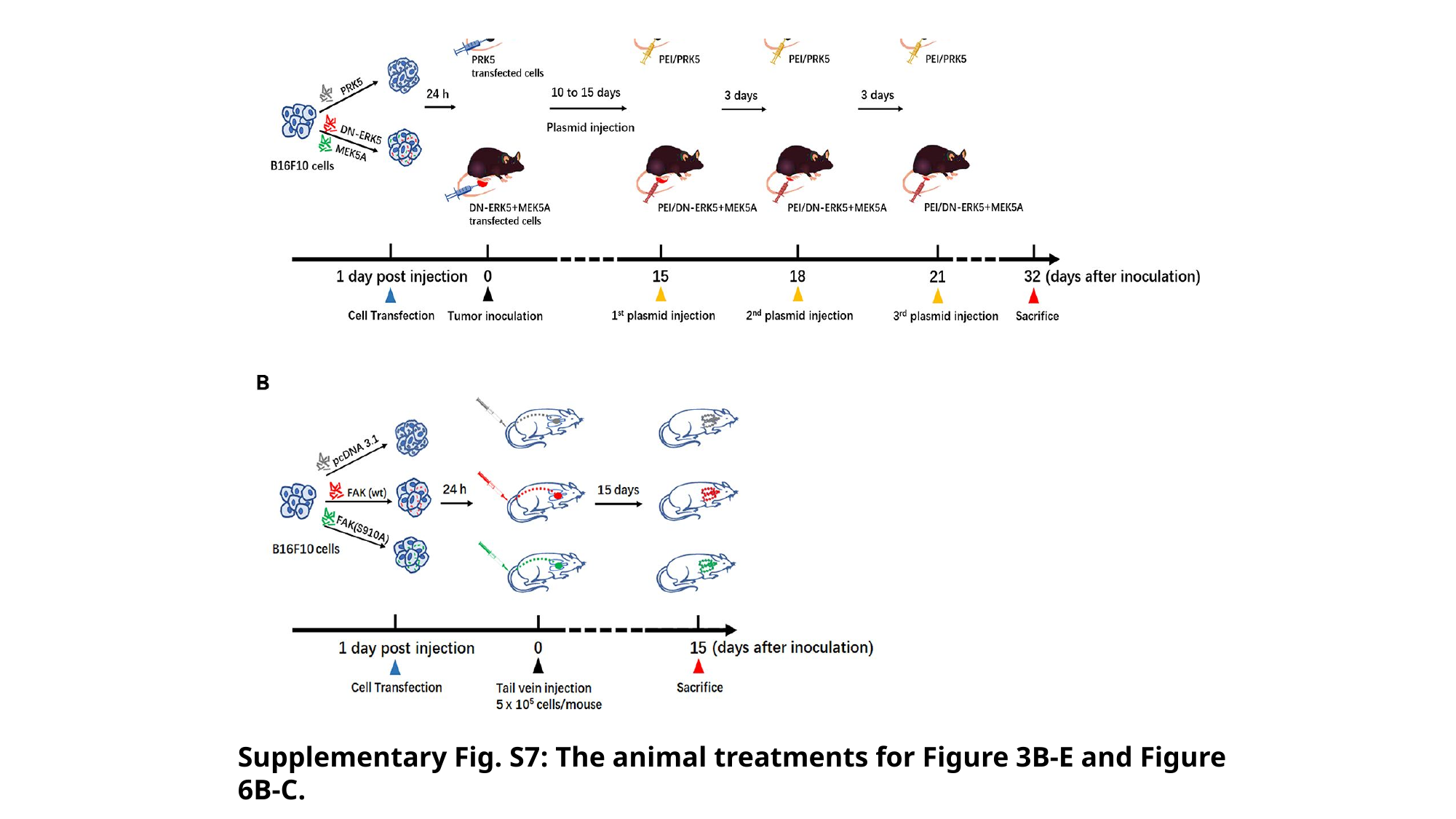

Supplementary Fig. S7: The animal treatments for Figure 3B-E and Figure 6B-C.

## Slide 8
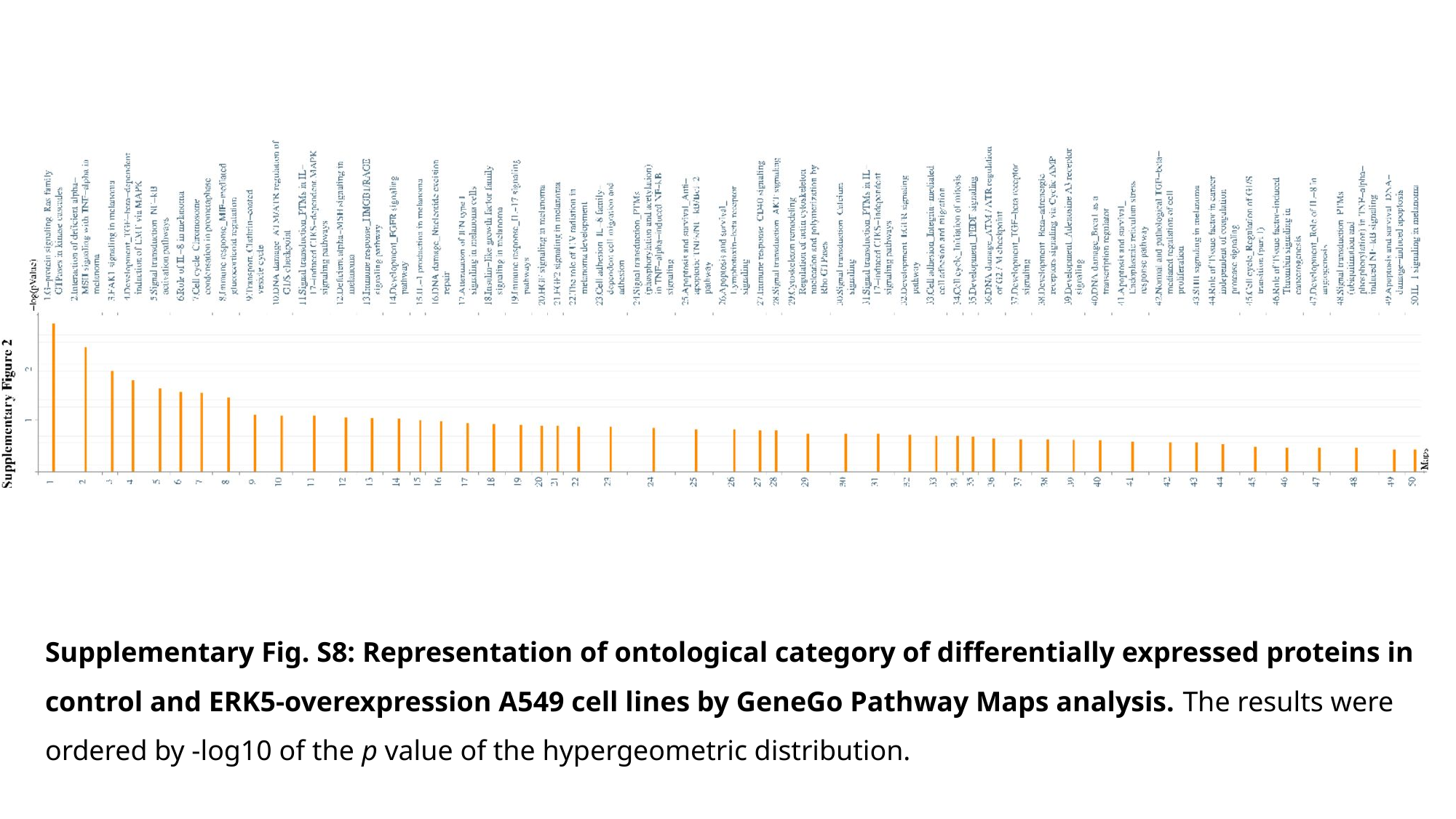

Supplementary Fig. S8: Representation of ontological category of differentially expressed proteins in control and ERK5-overexpression A549 cell lines by GeneGo Pathway Maps analysis. The results were ordered by -log10 of the p value of the hypergeometric distribution.

## Slide 9
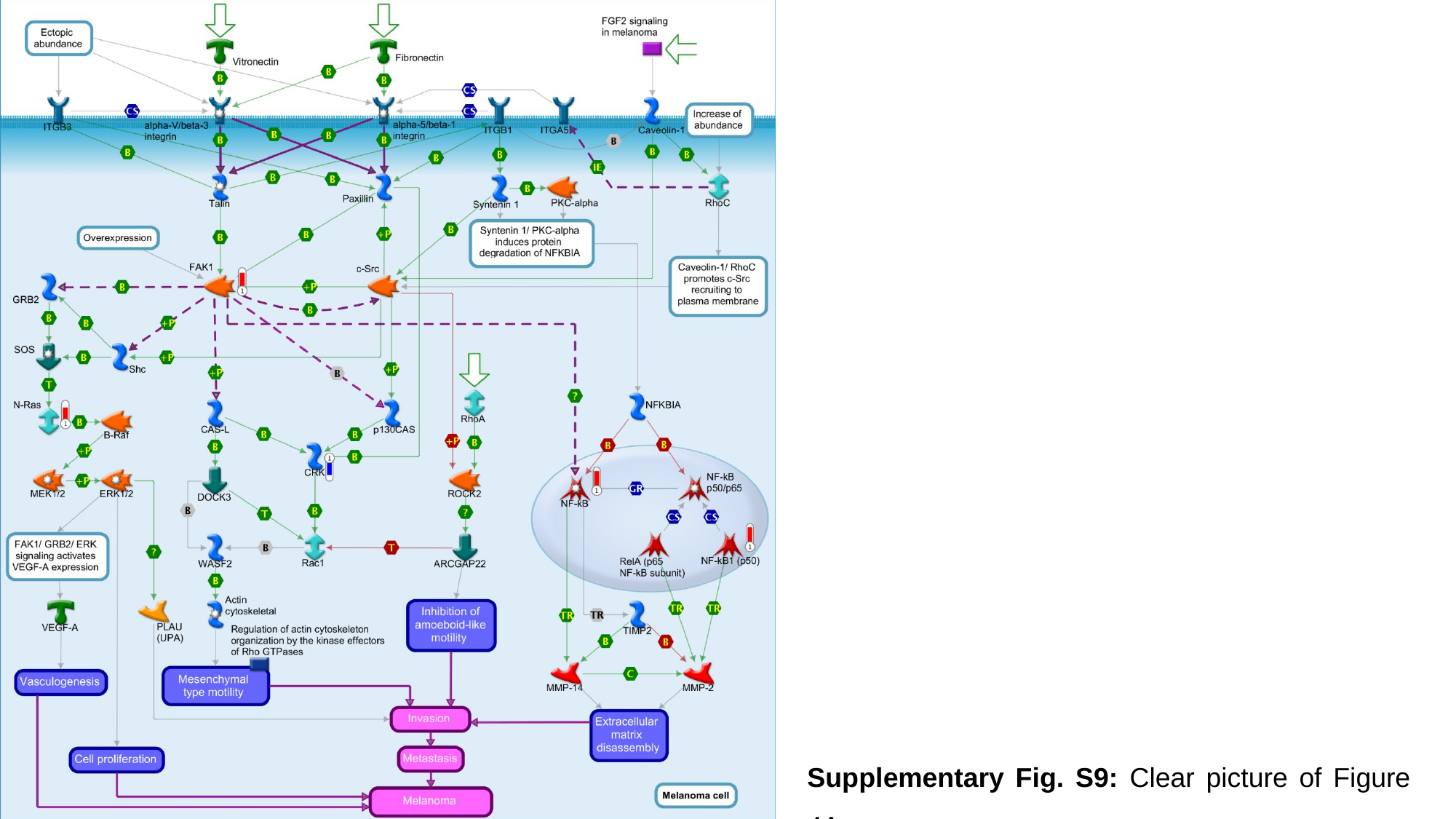

Supplementary Fig. S9: Clear picture of Figure 4A.

## Slide 10
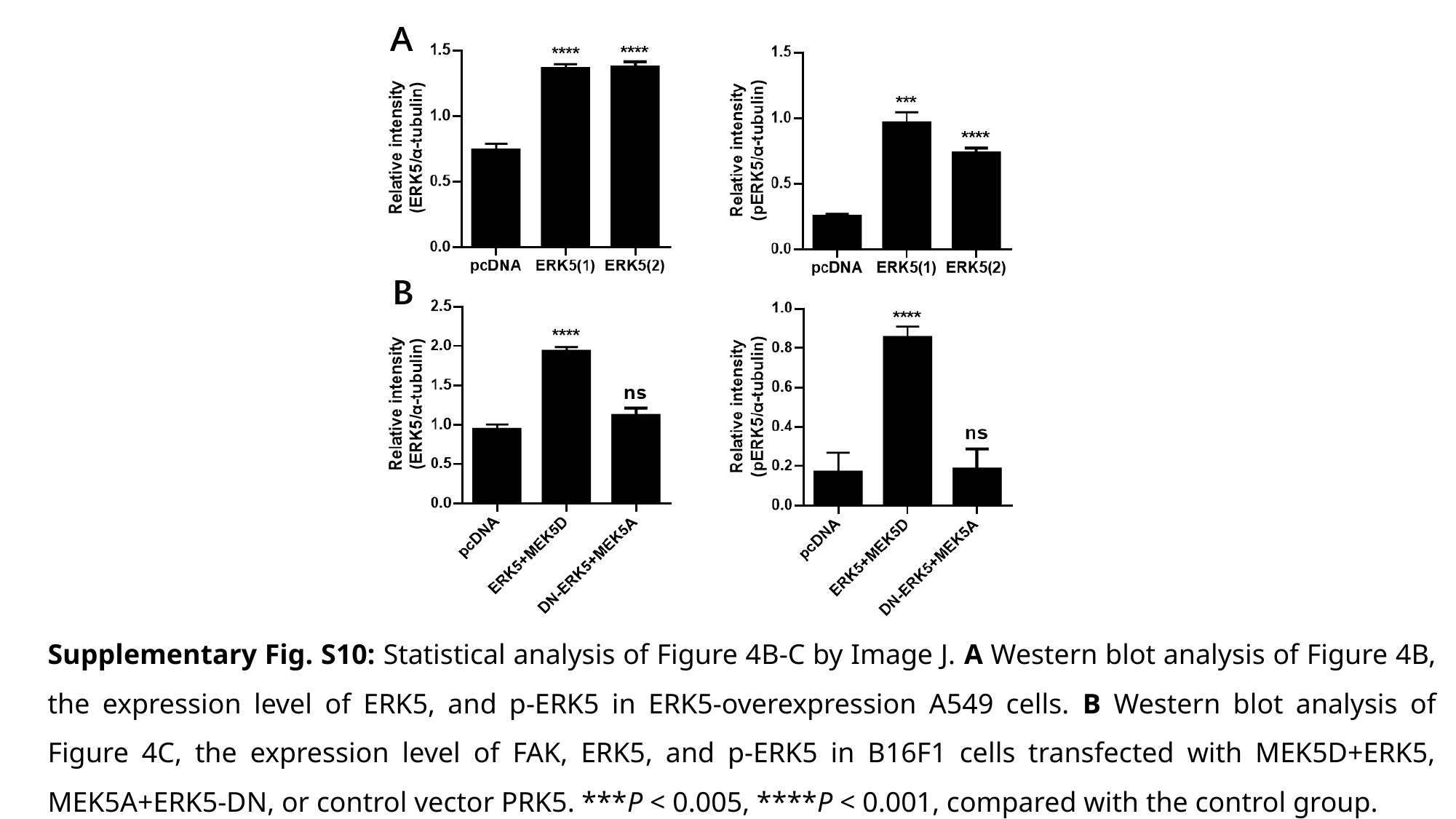

Supplementary Fig. S10: Statistical analysis of Figure 4B-C by Image J. A Western blot analysis of Figure 4B, the expression level of ERK5, and p-ERK5 in ERK5-overexpression A549 cells. B Western blot analysis of Figure 4C, the expression level of FAK, ERK5, and p-ERK5 in B16F1 cells transfected with MEK5D+ERK5, MEK5A+ERK5-DN, or control vector PRK5. ***P < 0.005, ****P < 0.001, compared with the control group.

## Slide 11
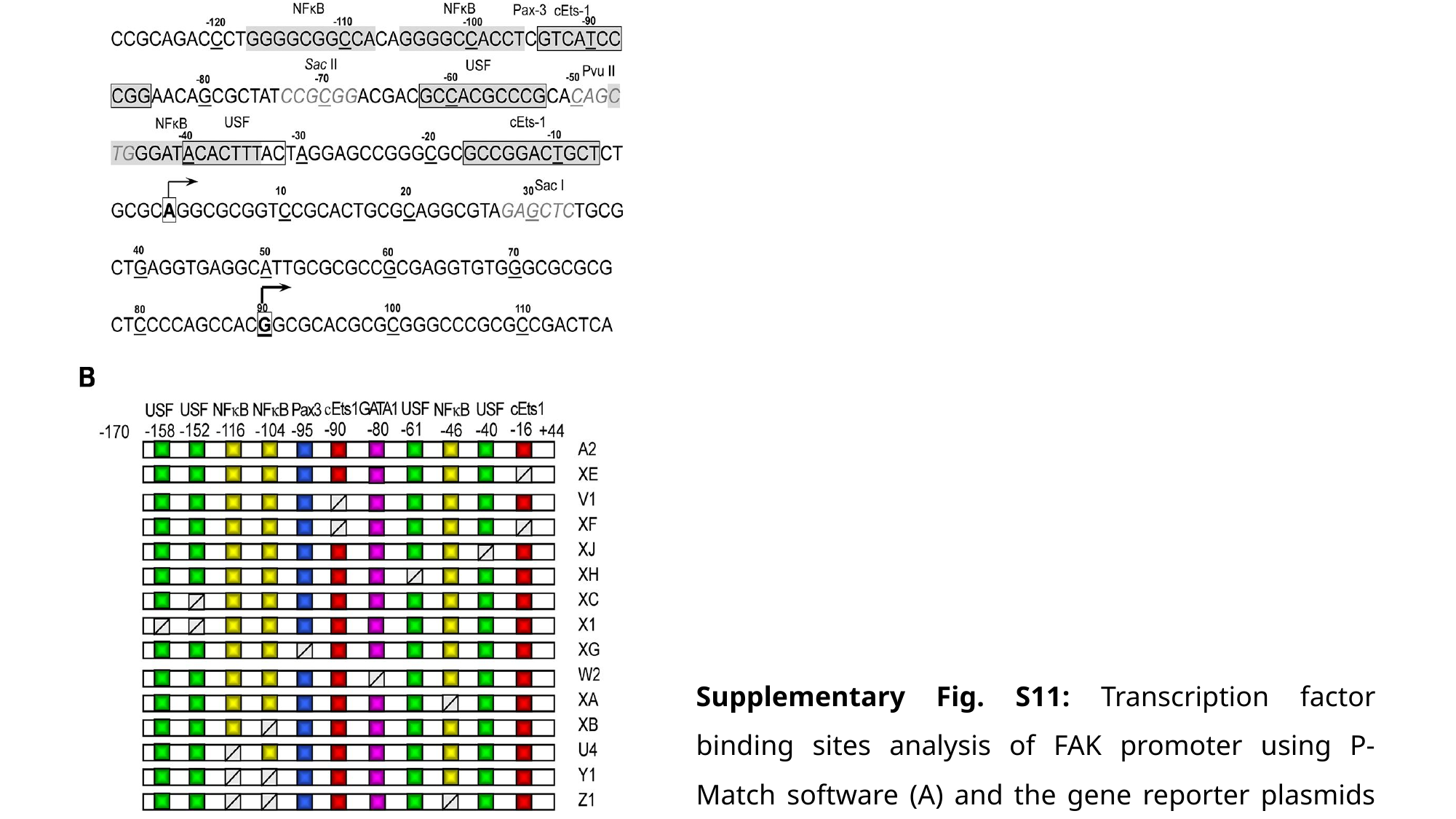

Supplementary Fig. S11: Transcription factor binding sites analysis of FAK promoter using P-Match software (A) and the gene reporter plasmids used in the study (B).

## Slide 12
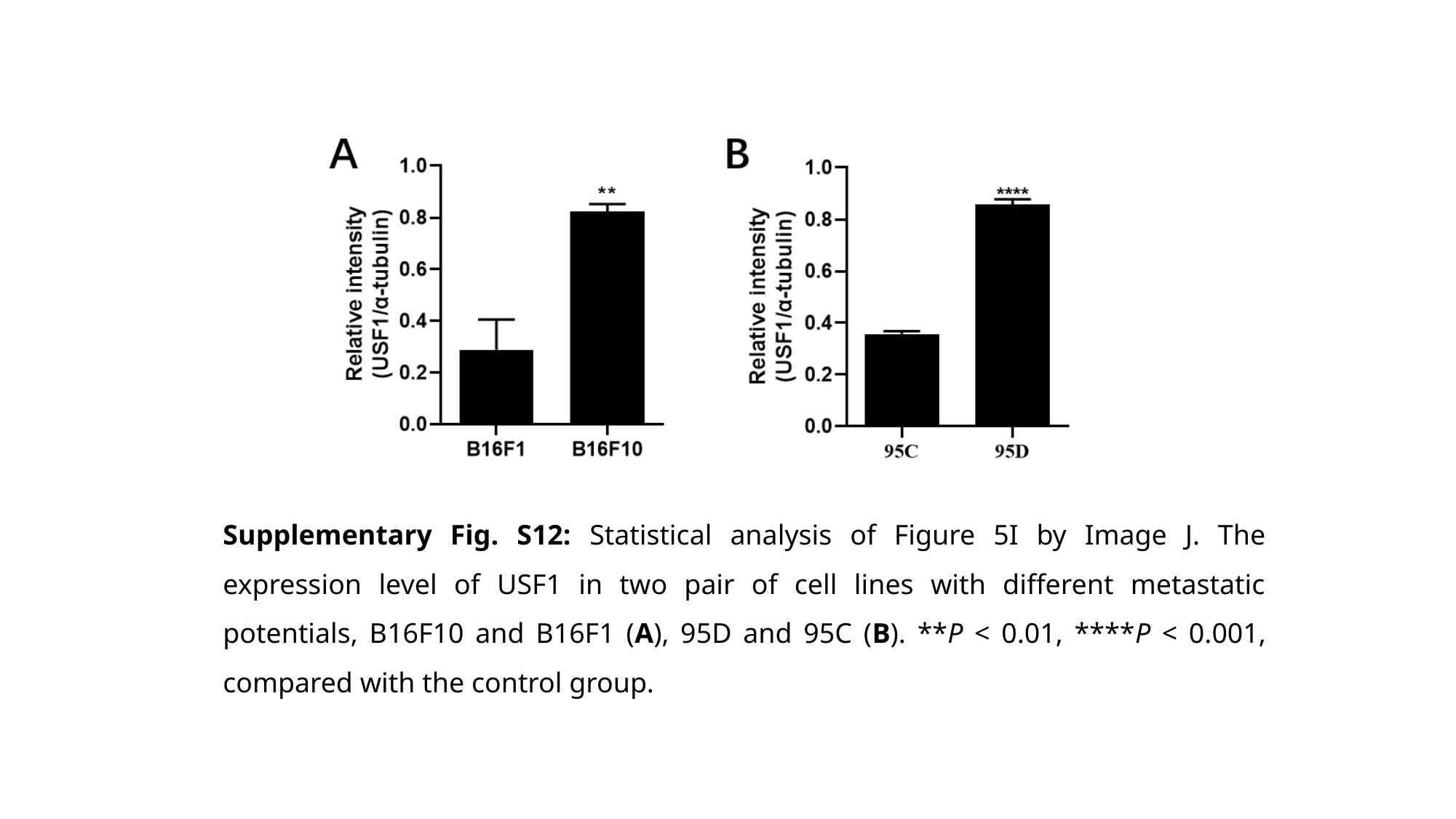

Supplementary Fig. S12: Statistical analysis of Figure 5I by Image J. The expression level of USF1 in two pair of cell lines with different metastatic potentials, B16F10 and B16F1 (A), 95D and 95C (B). **P < 0.01, ****P < 0.001, compared with the control group.

## Slide 13
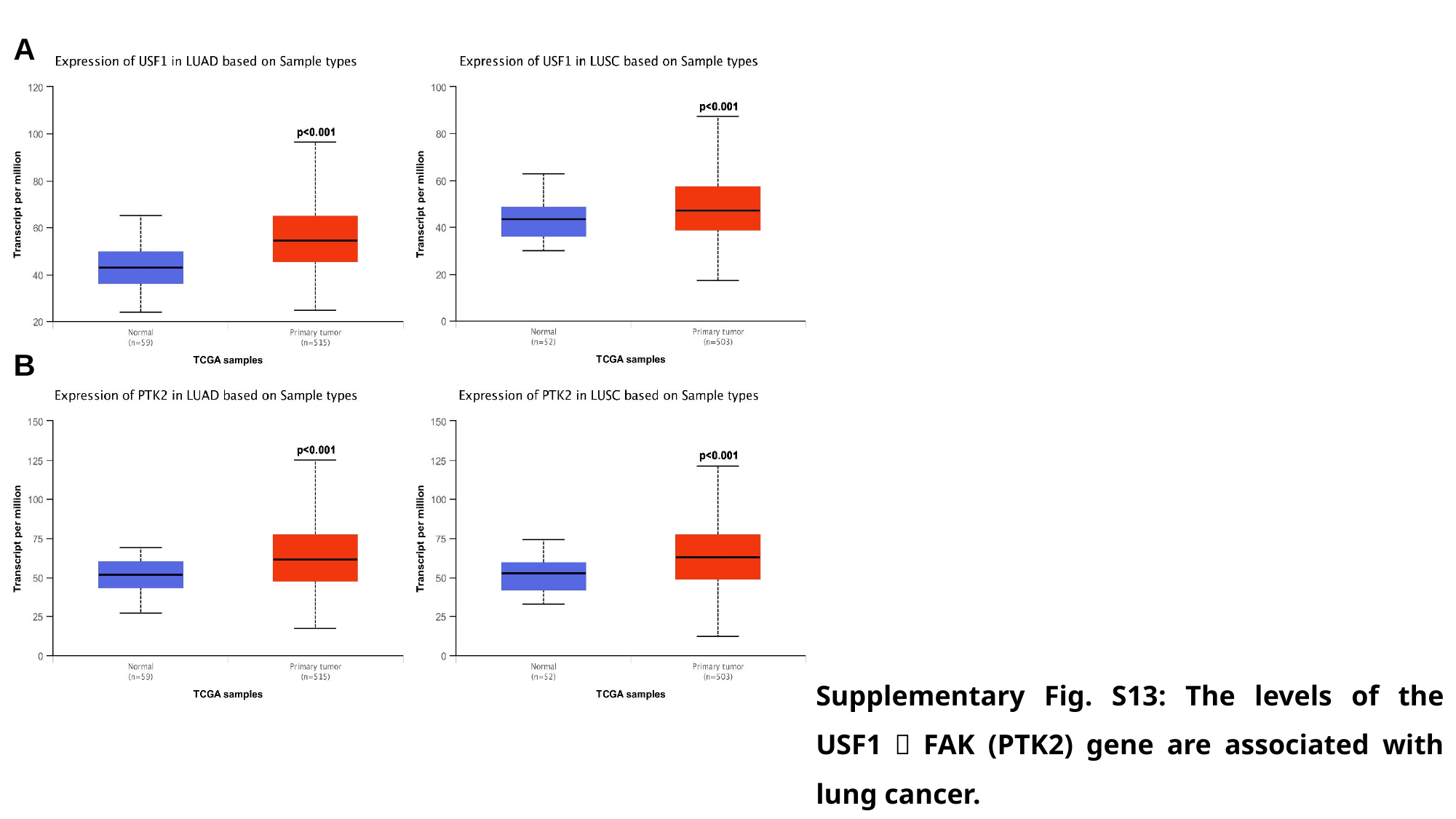

A
B
Supplementary Fig. S13: The levels of the USF1，FAK (PTK2) gene are associated with lung cancer.

## Slide 14
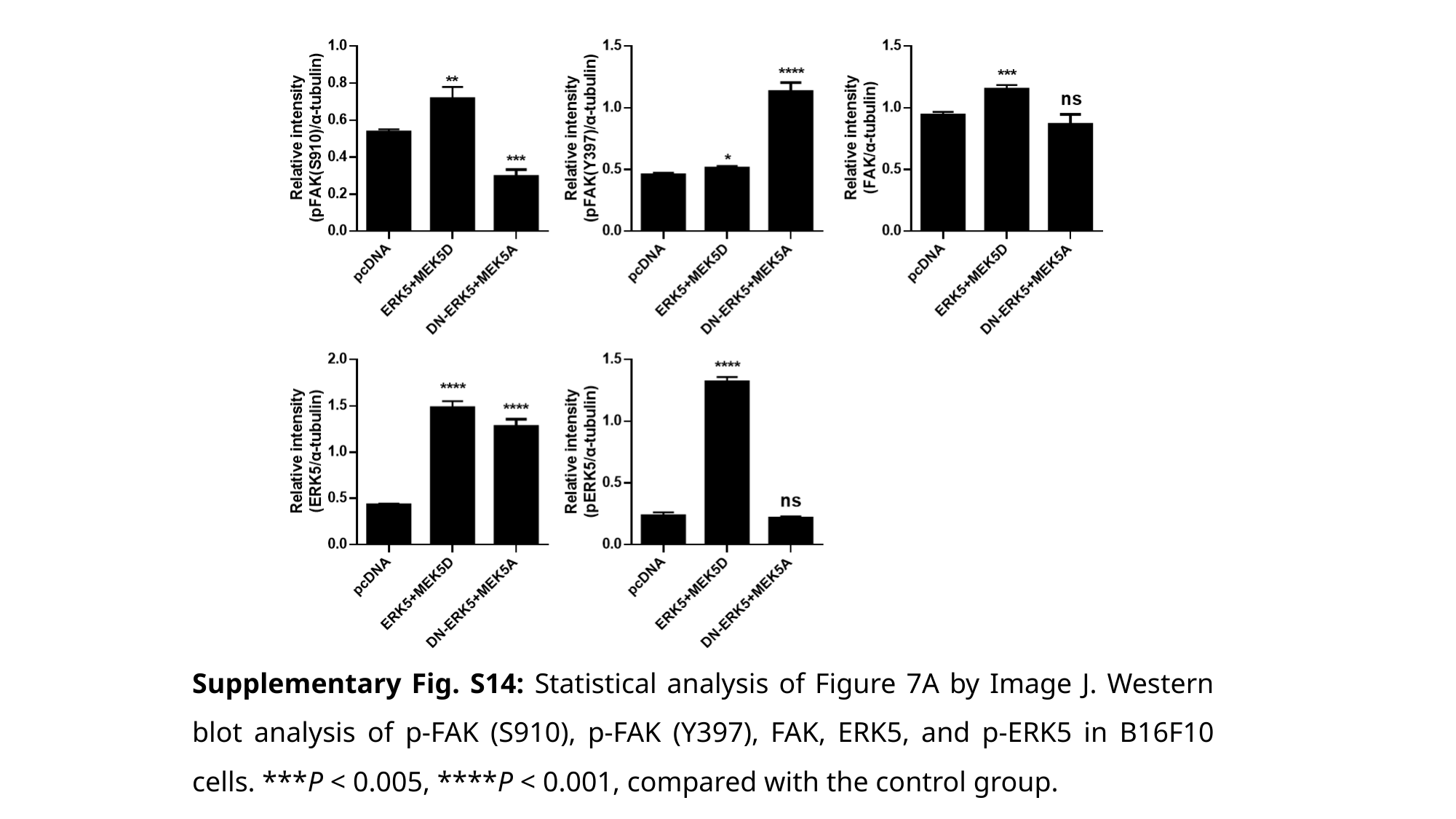

Supplementary Fig. S14: Statistical analysis of Figure 7A by Image J. Western blot analysis of p-FAK (S910), p-FAK (Y397), FAK, ERK5, and p-ERK5 in B16F10 cells. ***P < 0.005, ****P < 0.001, compared with the control group.

## Slide 15
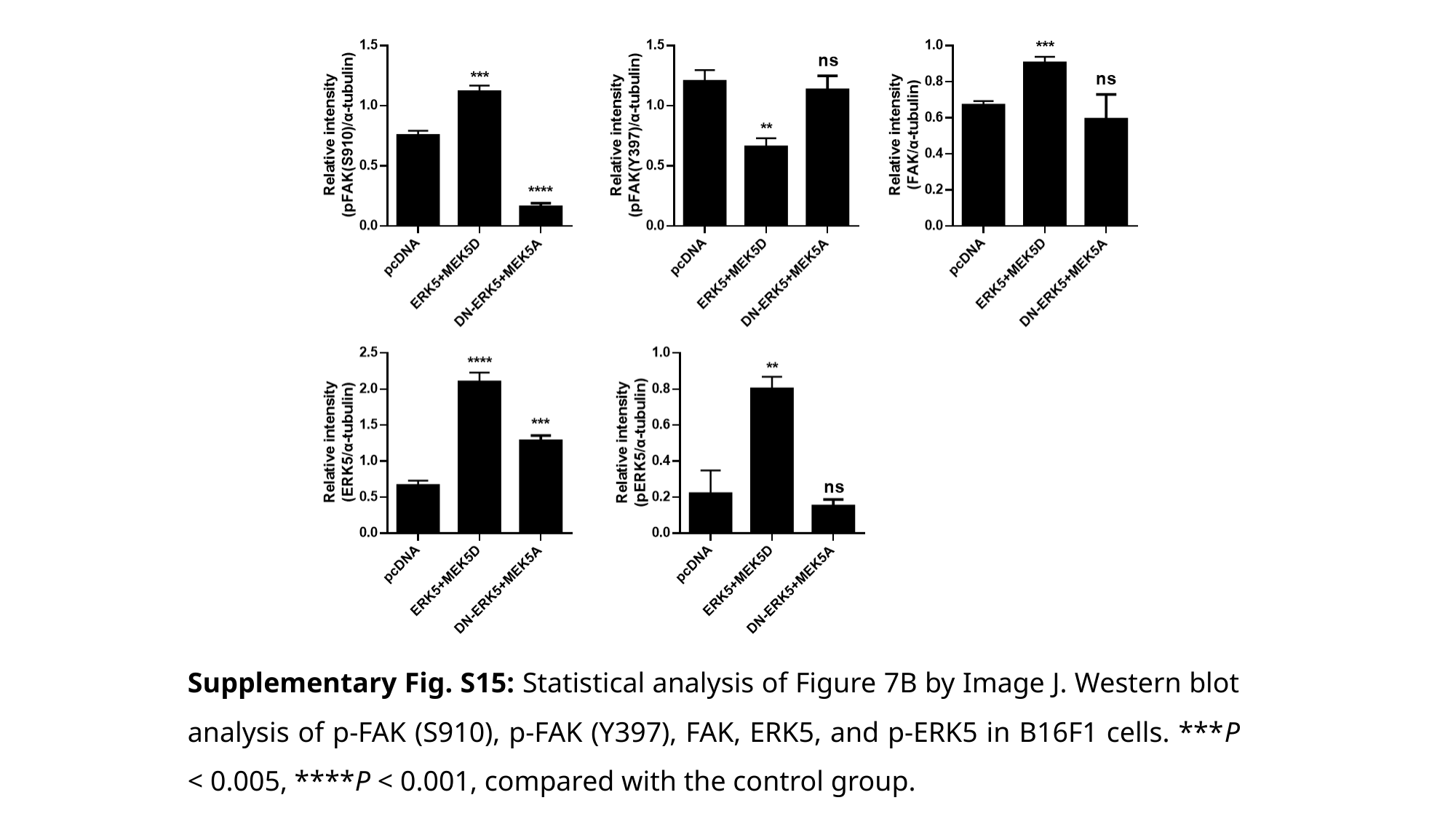

Supplementary Fig. S15: Statistical analysis of Figure 7B by Image J. Western blot analysis of p-FAK (S910), p-FAK (Y397), FAK, ERK5, and p-ERK5 in B16F1 cells. ***P < 0.005, ****P < 0.001, compared with the control group.

## Slide 16
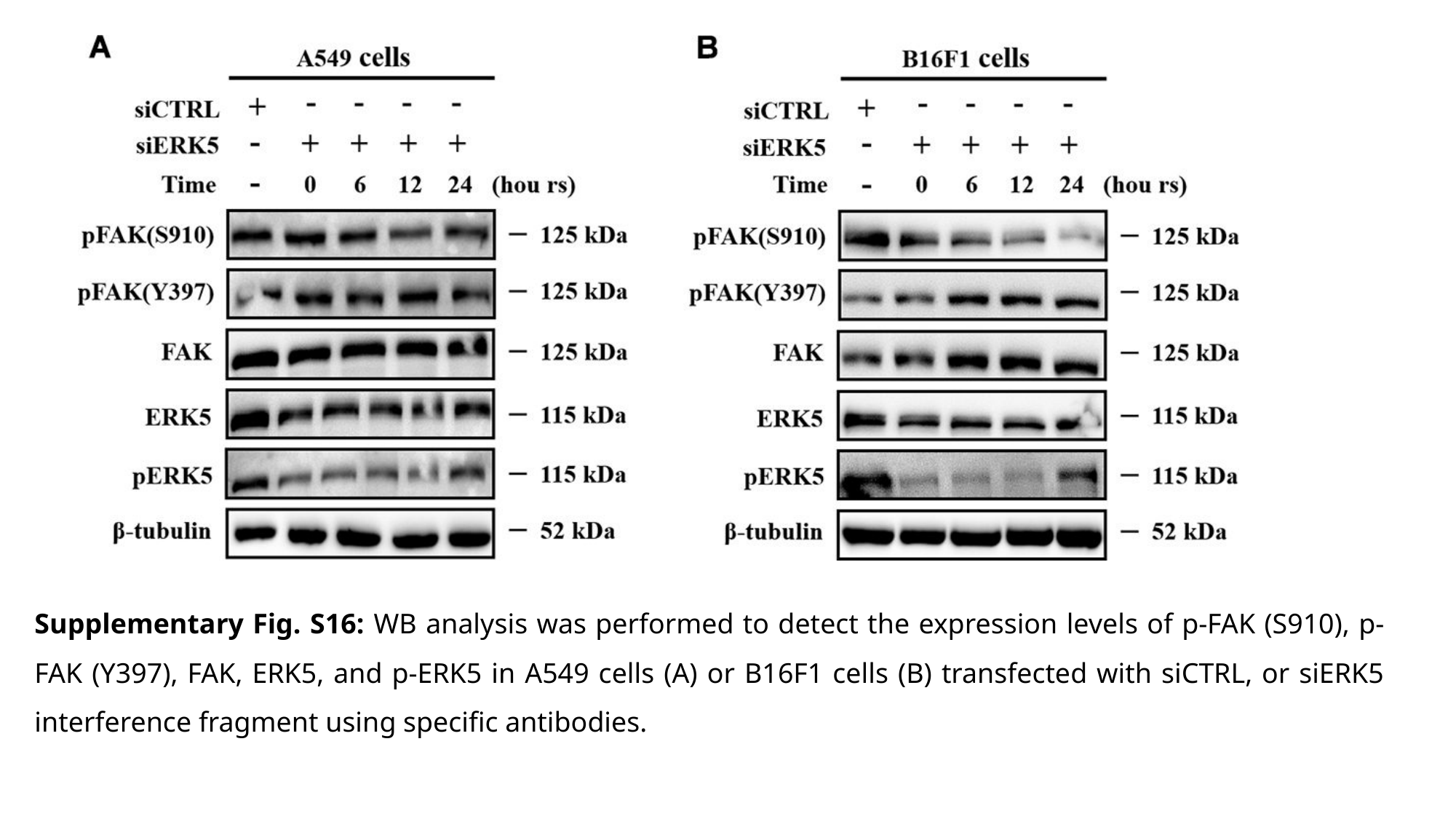

Supplementary Fig. S16: WB analysis was performed to detect the expression levels of p-FAK (S910), p-FAK (Y397), FAK, ERK5, and p-ERK5 in A549 cells (A) or B16F1 cells (B) transfected with siCTRL, or siERK5 interference fragment using specific antibodies.

## Slide 17
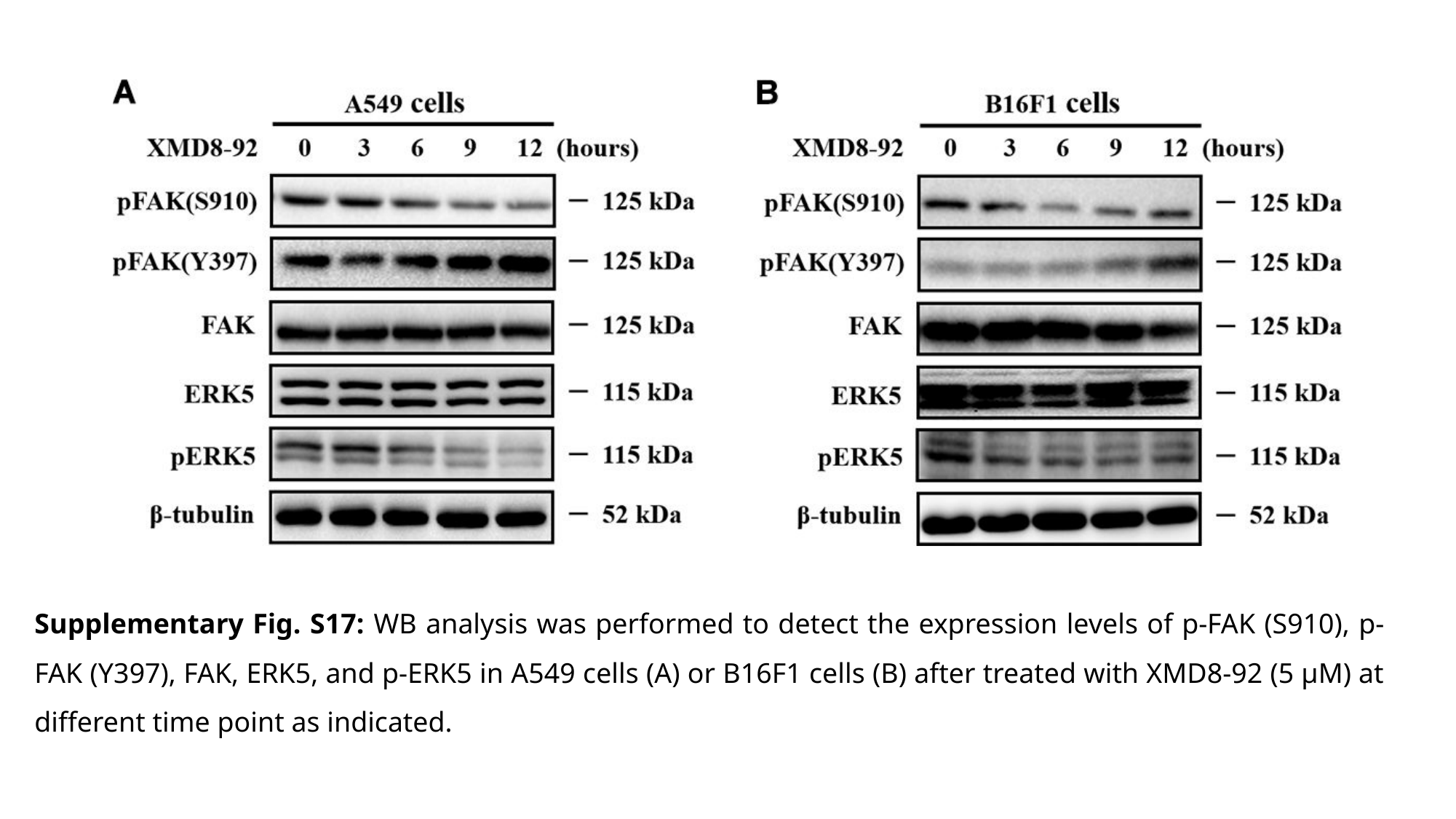

Supplementary Fig. S17: WB analysis was performed to detect the expression levels of p-FAK (S910), p-FAK (Y397), FAK, ERK5, and p-ERK5 in A549 cells (A) or B16F1 cells (B) after treated with XMD8-92 (5 μM) at different time point as indicated.

## Slide 18
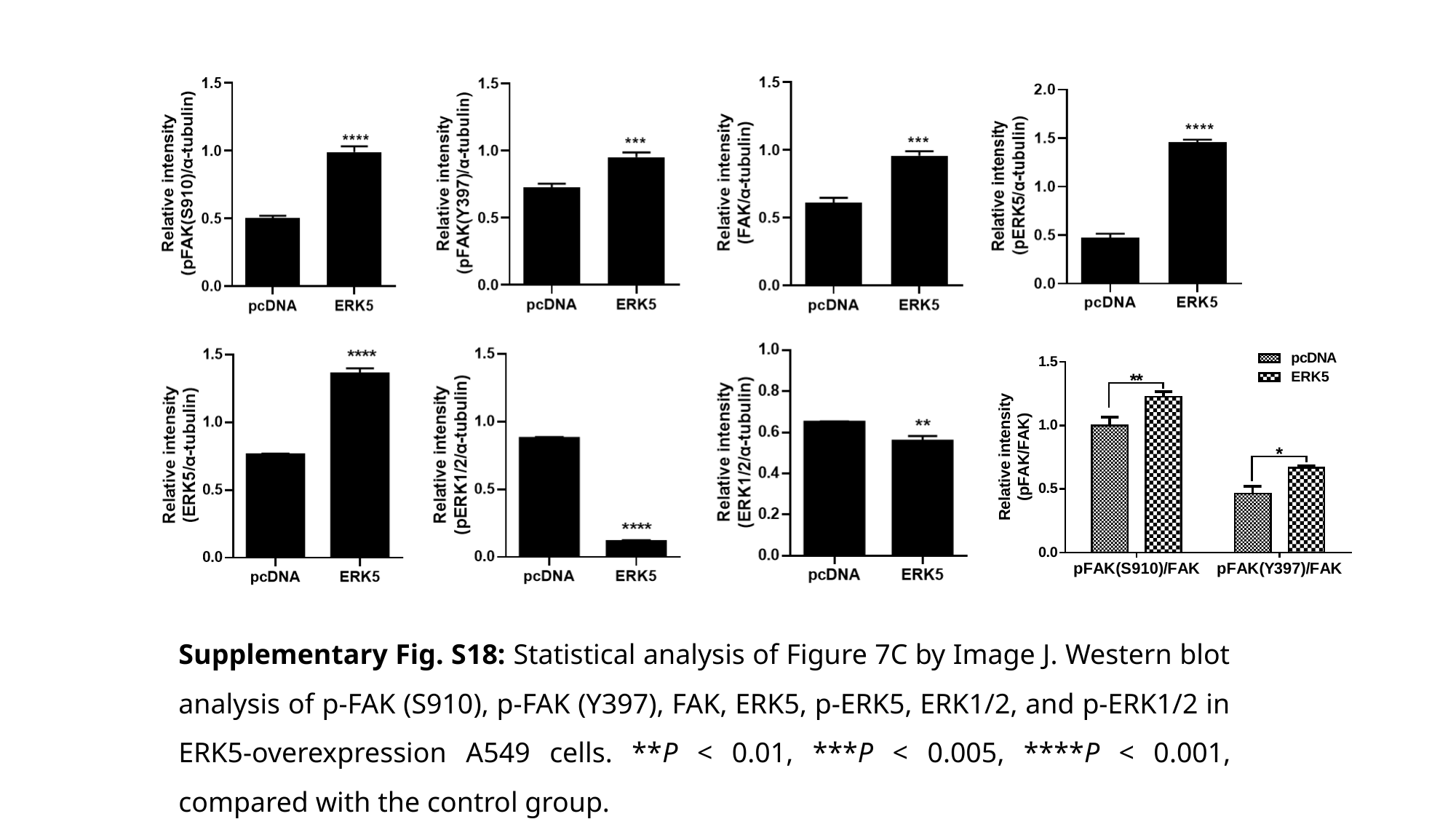

Supplementary Fig. S18: Statistical analysis of Figure 7C by Image J. Western blot analysis of p-FAK (S910), p-FAK (Y397), FAK, ERK5, p-ERK5, ERK1/2, and p-ERK1/2 in ERK5-overexpression A549 cells. **P < 0.01, ***P < 0.005, ****P < 0.001, compared with the control group.

## Slide 19
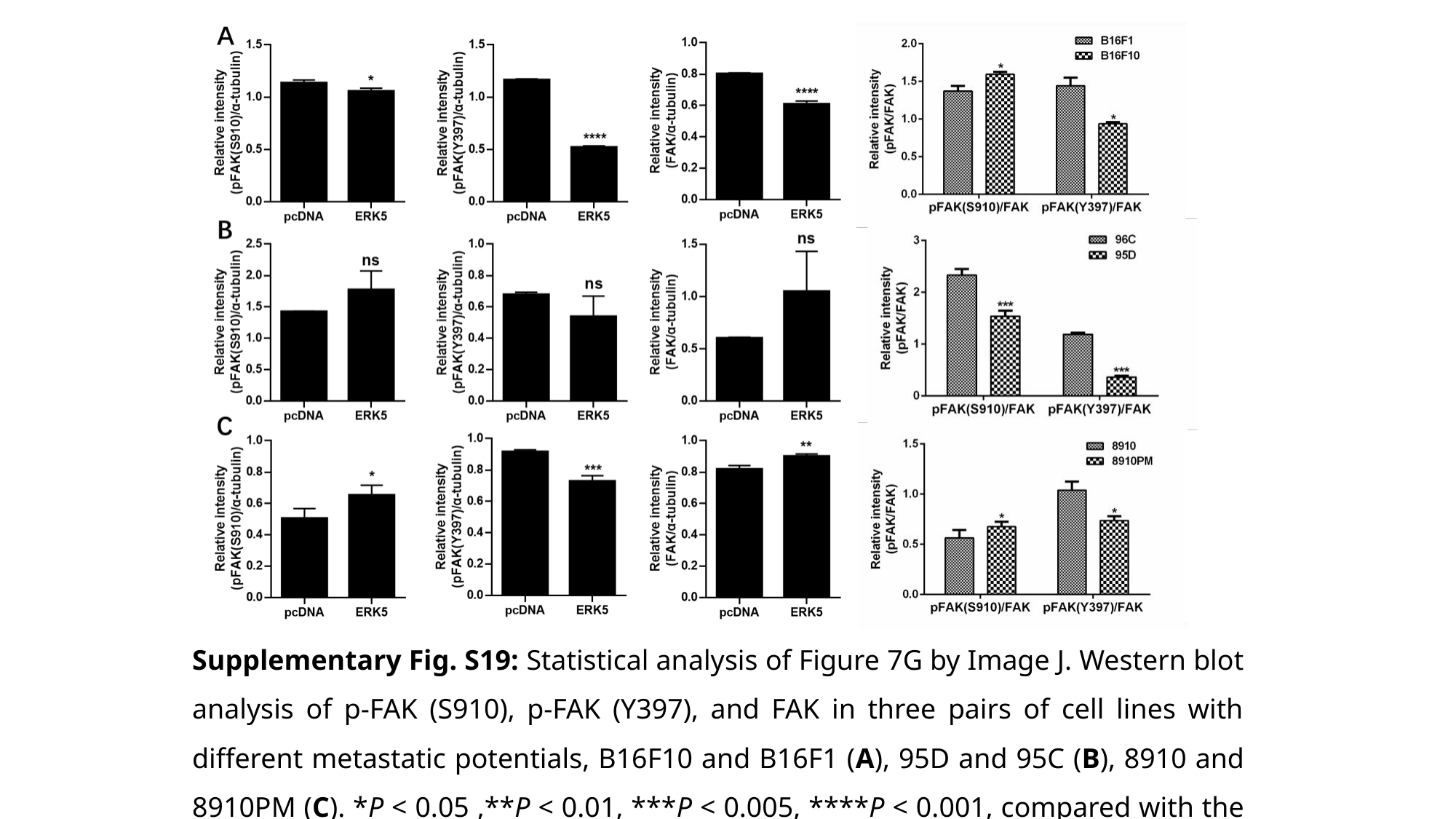

Supplementary Fig. S19: Statistical analysis of Figure 7G by Image J. Western blot analysis of p-FAK (S910), p-FAK (Y397), and FAK in three pairs of cell lines with different metastatic potentials, B16F10 and B16F1 (A), 95D and 95C (B), 8910 and 8910PM (C). *P < 0.05 ,**P < 0.01, ***P < 0.005, ****P < 0.001, compared with the control group.

## Slide 20
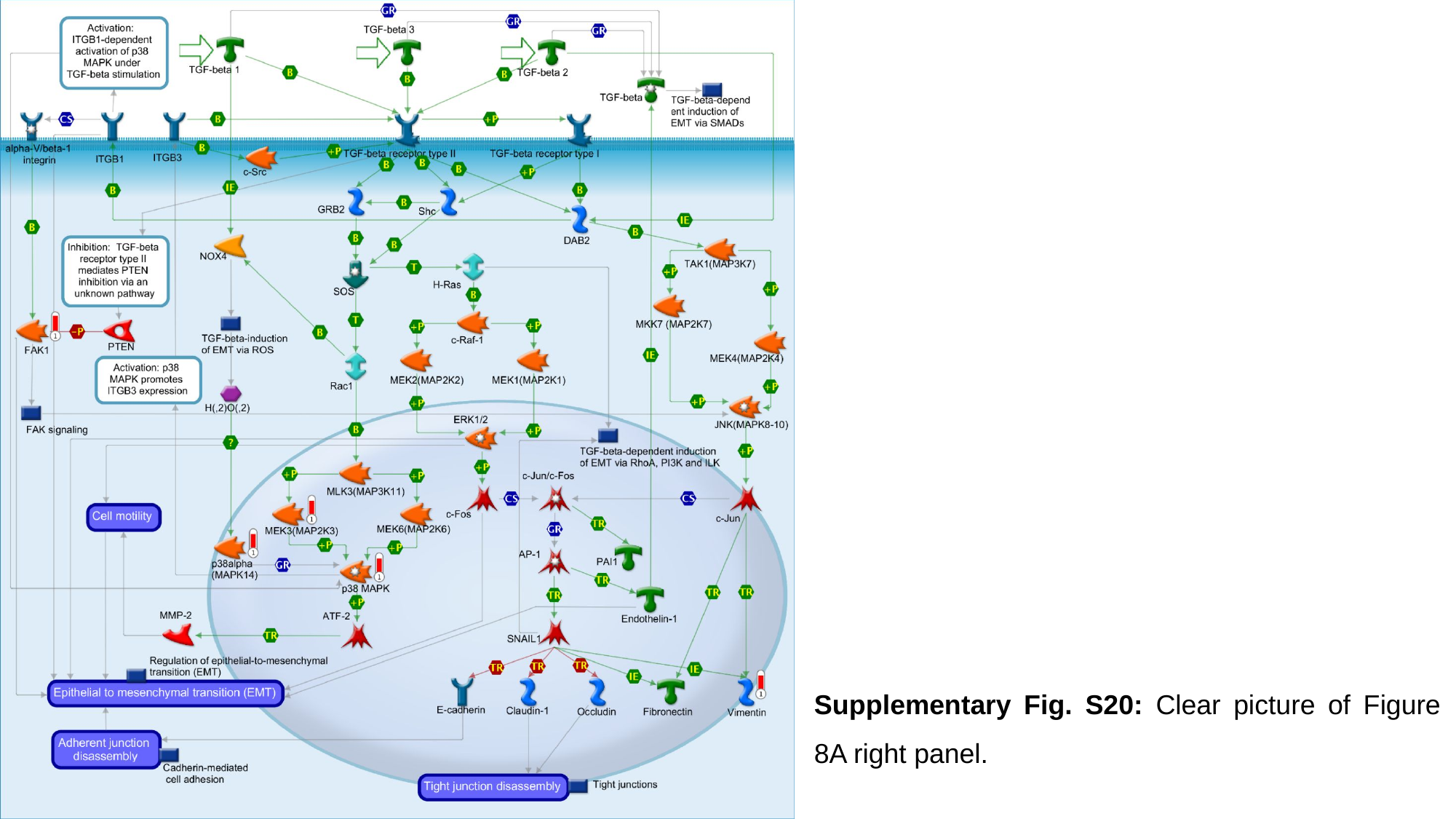

Supplementary Fig. S20: Clear picture of Figure 8A right panel.

## Slide 21
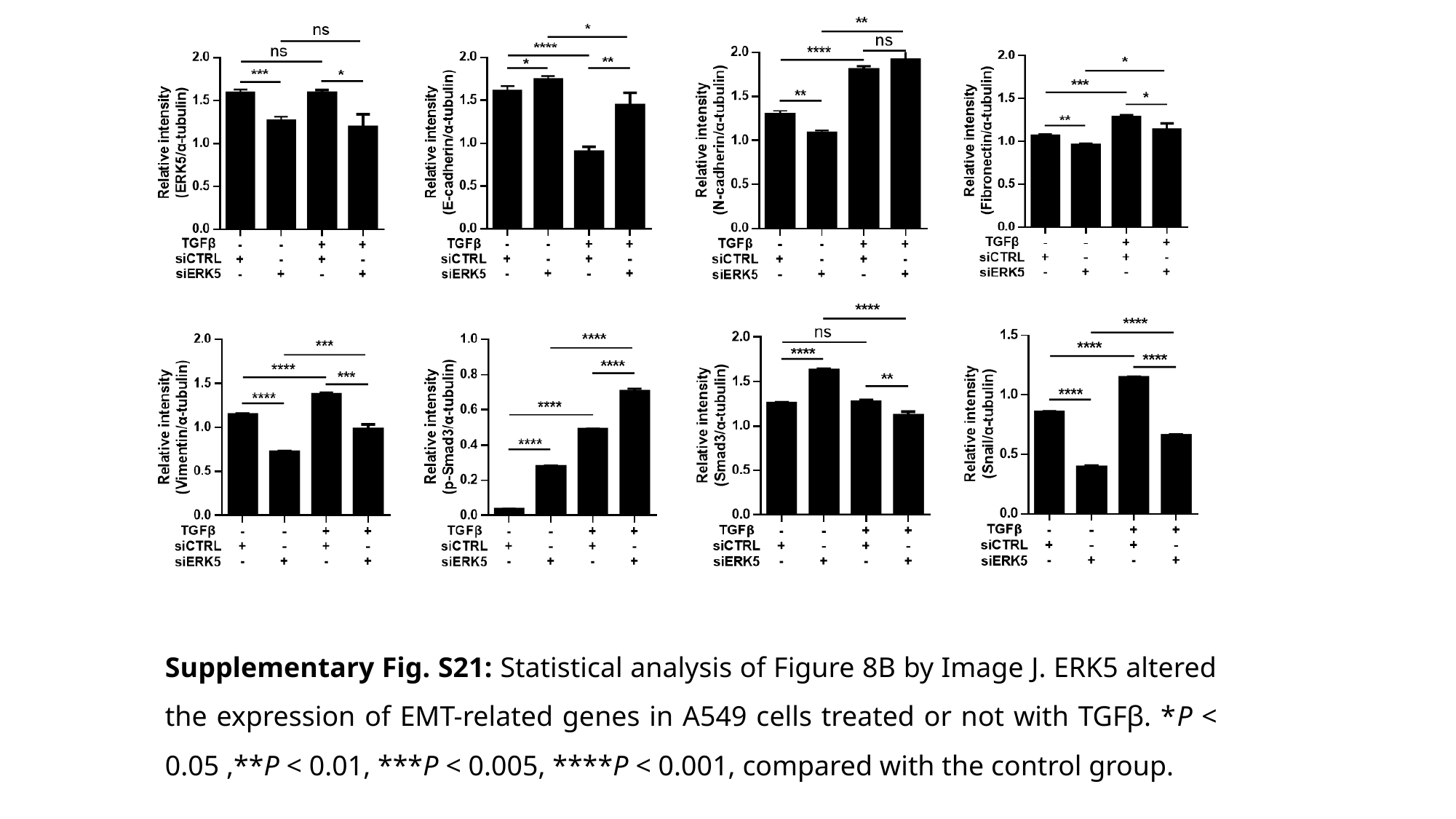

Supplementary Fig. S21: Statistical analysis of Figure 8B by Image J. ERK5 altered the expression of EMT-related genes in A549 cells treated or not with TGFβ. *P < 0.05 ,**P < 0.01, ***P < 0.005, ****P < 0.001, compared with the control group.

## Slide 22
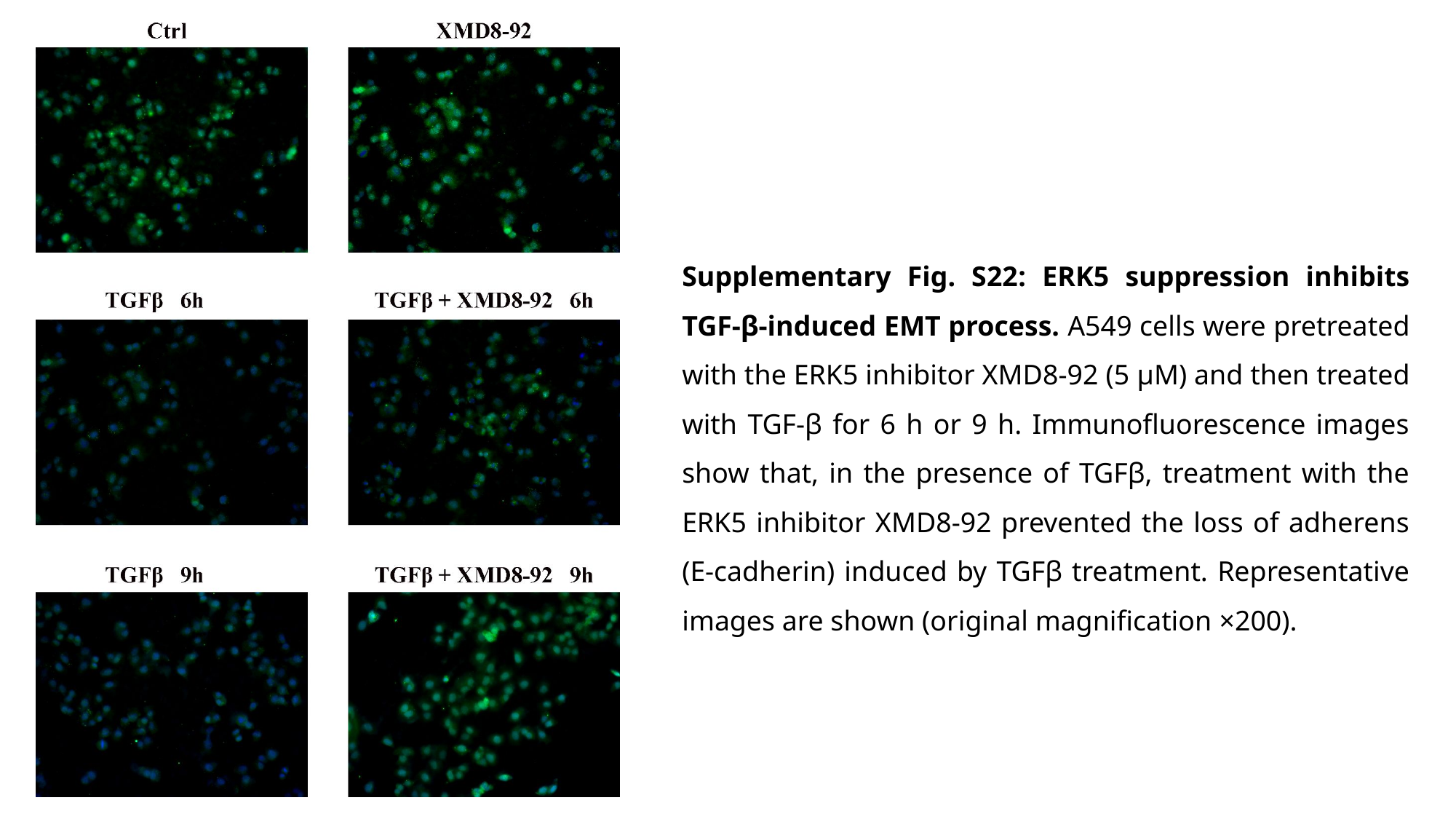

Supplementary Fig. S22: ERK5 suppression inhibits TGF-β-induced EMT process. A549 cells were pretreated with the ERK5 inhibitor XMD8-92 (5 μM) and then treated with TGF-β for 6 h or 9 h. Immunofluorescence images show that, in the presence of TGFβ, treatment with the ERK5 inhibitor XMD8-92 prevented the loss of adherens (E-cadherin) induced by TGFβ treatment. Representative images are shown (original magnification ×200).

## Slide 23
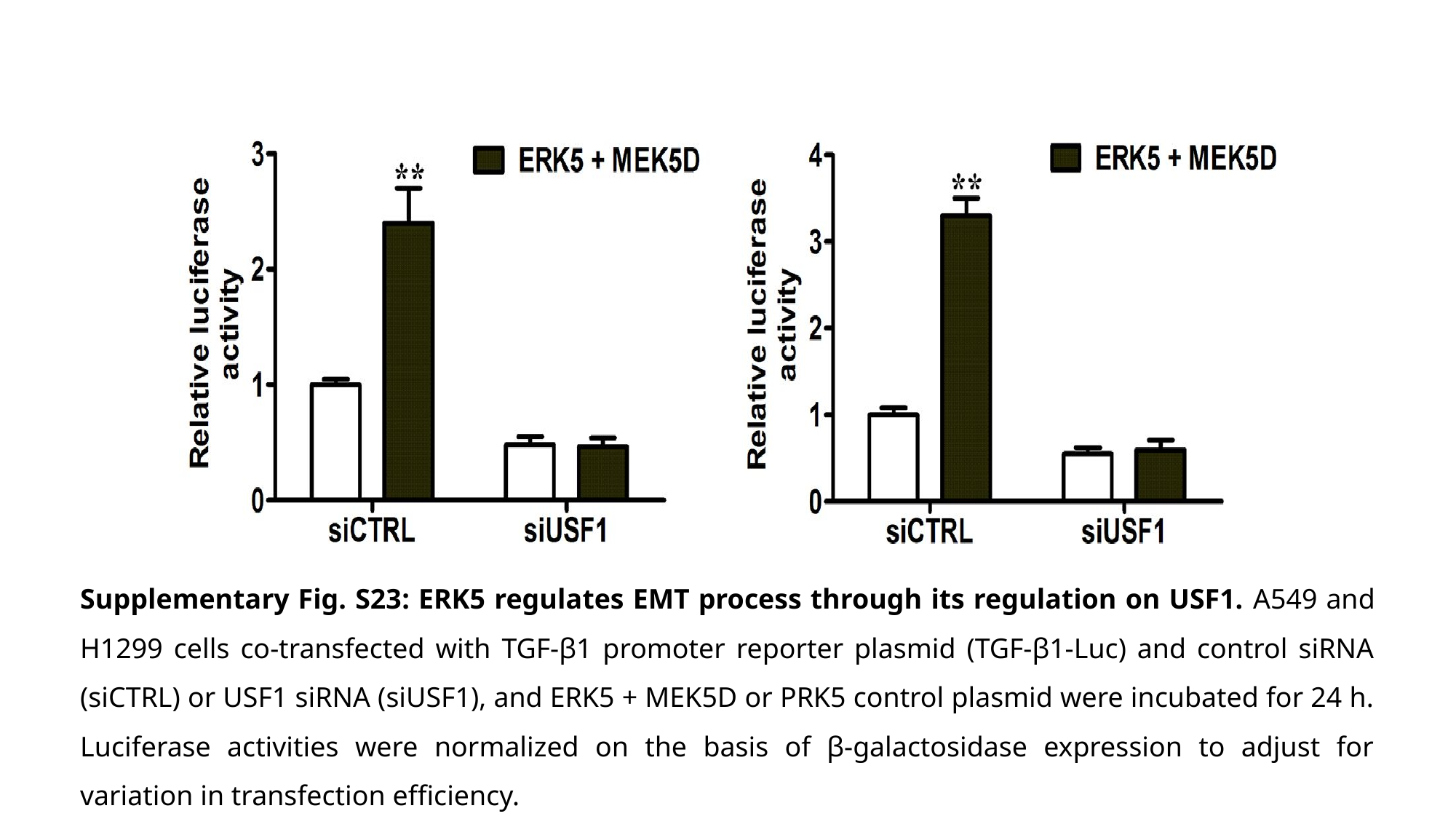

Supplementary Fig. S23: ERK5 regulates EMT process through its regulation on USF1. A549 and H1299 cells co-transfected with TGF-β1 promoter reporter plasmid (TGF-β1-Luc) and control siRNA (siCTRL) or USF1 siRNA (siUSF1), and ERK5 + MEK5D or PRK5 control plasmid were incubated for 24 h. Luciferase activities were normalized on the basis of β-galactosidase expression to adjust for variation in transfection efficiency.
