# Supplementary material for: Extracellular signal regulated kinase 5 promotes cell migration, invasion and lung metastasis in a FAK-dependent manner
Source: Protein Cell. 2020 Mar 6;11(11):825–45. doi: 10.1007/s13238-020-00701-1 (PMC7647985; doi:10.1007/s13238-020-00701-1)
Supplement: Supplementary file 3 — Supplementary material 3 (DOCX 14 kb) [file 13238_2020_701_MOESM3_ESM.docx]

**Table S1. The primers for variants construction of ERK5D, MEK5D and MEK5A.**

| Name | Upper primer (5’-3’) | Lower primer (5’-3’) |
| --- | --- | --- |
| ERK5D  (DNm) | acttcatgGctgagtTtgtggctactcgctggtac | caAactcagCcatgaagtactggtgctcggcag |
| MEK5D  (Dm) | atGAtatagccaagGACtatgttggaacaaatgcttac | aGTCcttggctataTCattcaccagctgtgtgctc |
| MEK5A (Am) | agcacacagctggtgaatGctatagccaagGcgtat | agcatttgttccaacatacgCcttggctatagCat |

**Table S2. The primers of selected genes for real time PCR.**

| Gene | Forward primer (5’-3’) | Reverse primer (5’-3’) |
| --- | --- | --- |
| E-cadherin | CGACCCTGCCTCTGAATCC | TACACGCTGGGAAACATGAGC |
| N-cadherin | CAATGACGTCCACCCTGTTCT | CTGCCATGACTTTCTACGGAGA |
| Fibronectin | CCCAGACTTATGGTGGCAATT | AATTTCCGCCTCGAGTCTGA |
| Vimentin | CCAACCTTTTCTTCCCTGAA | TTGAGTGGGTGTCAACCAGA |
| Snail | CTCTGAAGATGCACATCCGAA | GGCTTCTCACCAGTGTGGGT |
| MMP-2 | CCGTCGCCCATCATCAA | GGTATTGCACTGCCAACTCTTTG |
| β-Actin | TCCAGCCTTCCTTCTTGGGTATG | GAAGGTGGACAGTGAGGCCAGGAT |
